# Expanding the genomic encyclopedia of *Actinobacteria* with 824 isolate reference genomes

**DOI:** 10.1016/j.xgen.2022.100213

**Published:** 2022-11-11

**Authors:** Rekha Seshadri, Simon Roux, Katharina J. Huber, Dongying Wu, Sora Yu, Dan Udwary, Lee Call, Stephen Nayfach, Richard L. Hahnke, Rüdiger Pukall, James R. White, Neha J. Varghese, Cody Webb, Krishnaveni Palaniappan, Lorenz C. Reimer, Joaquim Sardà, Jonathon Bertsch, Supratim Mukherjee, T.B.K. Reddy, Patrick P. Hajek, Marcel Huntemann, I-Min A. Chen, Alex Spunde, Alicia Clum, Nicole Shapiro, Zong-Yen Wu, Zhiying Zhao, Yuguang Zhou, Lyudmila Evtushenko, Sofie Thijs, Vincent Stevens, Emiley A. Eloe-Fadrosh, Nigel J. Mouncey, Yasuo Yoshikuni, William B. Whitman, Hans-Peter Klenk, Tanja Woyke, Markus Göker, Nikos C. Kyrpides, Natalia N. Ivanova

**Affiliations:** 1 US Department of Energy Joint Genome Institute, Berkeley, CA, USA; 2 Leibniz Institute DSMZ - German Collection of Microorganisms and Cell Cultures, Braunschweig, Germany; 3 Environmental Genomics and Systems Biology Division, Lawrence Berkeley National Laboratory, Berkeley, CA, USA; 4 Resphera Biosciences, Baltimore, MD, USA; 5 China General Microbiological Culture Collection Center, Beijing, China; 6 Pushchino Scientific Center for Biological Research of the Russian Academy of Sciences, All-Russian Collection of Microorganisms (VKM), Pushchino, Russia; 7 Center for Environmental Sciences, Environmental Biology, Hasselt University, Diepenbeek, Belgium; 8 Department of Microbiology, University of Georgia, Athens, GA, USA; 9 School of Biology, Newcastle University, Newcastle upon Tyne, UK; 10 Biological Systems and Engineering Division, Lawrence Berkeley National Laboratory, Berkeley, CA 94720, USA; 11 Center for Advanced Bioenergy and Bioproducts Innovation, Lawrence Berkeley National Laboratory, Berkeley, CA 94720, USA; 12 Global Institution for Collaborative Research and Education, Hokkaido University, Hokkaido 060-8589, Japan

**Keywords:** microbiology, comparative genomics, secondary metabolites, actinobacteria, metagenomics, ecology, evolution, mycobacteria

## Abstract

The phylum *Actinobacteria* includes important human pathogens like *Mycobacterium tuberculosis* and *Corynebacterium diphtheriae* and renowned producers of secondary metabolites of commercial interest, yet only a small part of its diversity is represented by sequenced genomes. Here, we present 824 actinobacterial isolate genomes in the context of a phylum-wide analysis of 6,700 genomes including public isolates and metagenome-assembled genomes (MAGs). We estimate that only 30%–50% of projected actinobacterial phylogenetic diversity possesses genomic representation via isolates and MAGs. A comparison of gene functions reveals novel determinants of host-microbe interaction as well as environment-specific adaptations such as potential antimicrobial peptides. We identify plasmids and prophages across isolates and uncover extensive prophage diversity structured mainly by host taxonomy. Analysis of >80,000 biosynthetic gene clusters reveals that horizontal gene transfer and gene loss shape secondary metabolite repertoire across taxa. Our observations illustrate the essential role of and need for high-quality isolate genome sequences.

## Highlights

• 824 new actinobacterial isolate genomes from diverse environments
• Only a third of actinobacterial diversity has genome representation
• New niche-specific gene determinants highlighted, such as new antimicrobial peptides
• Secondary metabolite gene clusters shaped by horizontal gene transfer

## Graphical abstract

**Figure:**
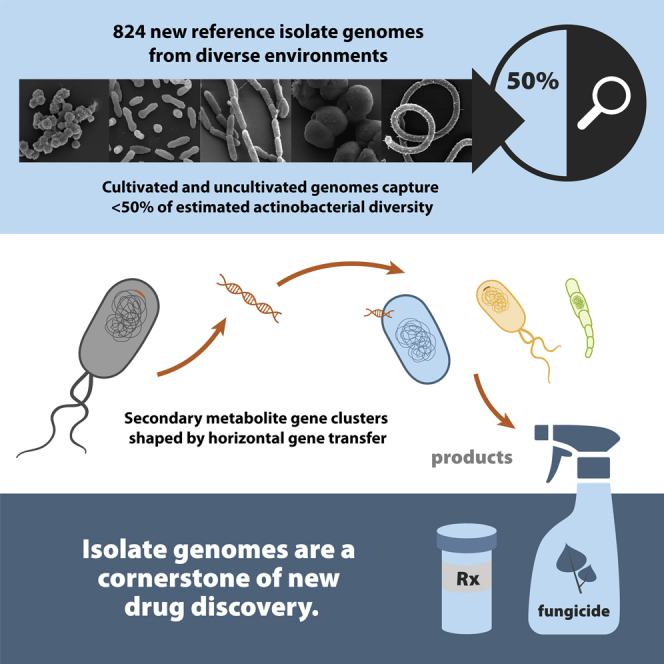

## Teaser

Seshadri et al. contribute 824 new genomes of cultivated *Actinobacteria*, which are important for drug discovery. They observe that the genes responsible for producing such compounds often move around between microbes, making them harder to capture without high-quality genomes. They highlight interesting adaptations such as an experimentally verified antimicrobial peptide.

## Introduction

*Actinobacteria* is a large and diverse phylum comprising Gram-positive bacteria with high guanine-plus-cytosine (G + C) genome content and genome sizes ranging from <0.5 to 15.0 Mbp. Members of this phylum exhibit varying morphological and physiological features, including multicellularity and complex differentiation and are widely (and abundantly) distributed in diverse ecosystems.^1^^,^^2^ Famous *Actinobacteria* include the causative agents of tuberculosis and diphtheria, some of the most devastating diseases in human history.^3^ Others play key ecological roles in carbon cycles of soil and aquatic environments or are widespread as mutualistic symbionts of plants and animals, synthesizing natural products for host benefit or helping herbivores digest plant biomass. As renowned producers of diverse secondary metabolites including over two-thirds of all antibiotics in current clinical use and other compounds of clinical or agricultural importance, they are the subject of numerous natural product discovery efforts.^1^^,^^4^^,^^5^^,^^6^^,^^7^

Despite their significance, *Actinobacteria* represent <10% of the 200,000+ publicly available genomes to date, and even these belong primarily to organisms relevant to human and veterinary medicine.^8^ As of January 2020 (analysis start date), 18,411 actinobacterial isolate genomes were available in public databases, although a considerable proportion belonged to multiple strains of human pathogens like *Mycobacterium tuberculosis* and *Mycobacteroides abscessus*.

In this study, we report the genomes of 824 actinobacterial isolates sequenced under the auspices of the Genomic Encyclopedia of Bacteria and Archaea (GEBA) initiative,^9^ mostly of type strains from the Leibniz Institute DSMZ culture collection sourced from diverse habitats. Type strains are permanently attached to the names of species and subspecies as regulated by the International Code of Nomenclature of Prokaryotes (ICNP),^10^ are well characterized with regard to phenotype, isolation sources, and other criteria, and have been made available to the worldwide scientific community via at least two different culture collections. A saturated collection of reference genomes of such isolates with pre-existing biochemical and genetic characterization (e.g., BacDive^11^) serves as a solid foundation for an array of experiments, including the development of microbial model systems and analyses of biotechnologically relevant pathways. Also, new opportunity for comparisons with non-pathogenic relatives could yield new insights and gene targets, expanding our understanding of important actinobacterial pathogens.

Here, we undertook a phylum-wide comparative analysis combining the 824 newly sequenced genomes with 5,922 non-redundant public actinobacterial genomes to explore (1) the overall phylogenetic diversity and cultivation status of the phylum, (2) niche-specific functional adaptations of different representatives, and (3) a compendium of natural product-encoding biosynthetic gene clusters (BGCs) and the drivers of that diversity. The data and comprehensive analyses generated herein are of broad utility in the fields of biological, biomedical, agricultural, and environmental sciences.

## Results and discussion

### Description of study datasets

A total of 824 high-quality draft genomes of isolates of the phylum *Actinobacteria*^12^ were sequenced, assembled, and annotated (>99.33% average [avg.] completeness, <1.36% avg. contamination, 1.88 Mbp avg. scaffold N50; see STAR Methods and Table S1). We chose to retain the phylum name *Actinobacteria* due to its familiarity to a broad readership but revised phylum names include *Actinobacteriota* and *Actinomycetota,* with this latter name being recently validly published.^13^ These genomes (hereafter referred to as “GEBA-Actino”) were processed using the IMG annotation pipeline,^14^ resulting in 4,569,551 predicted coding sequences from over 4.9 Gbp assembled sequence data (see Table S1 for complete list with metadata).

**Figure 1:**
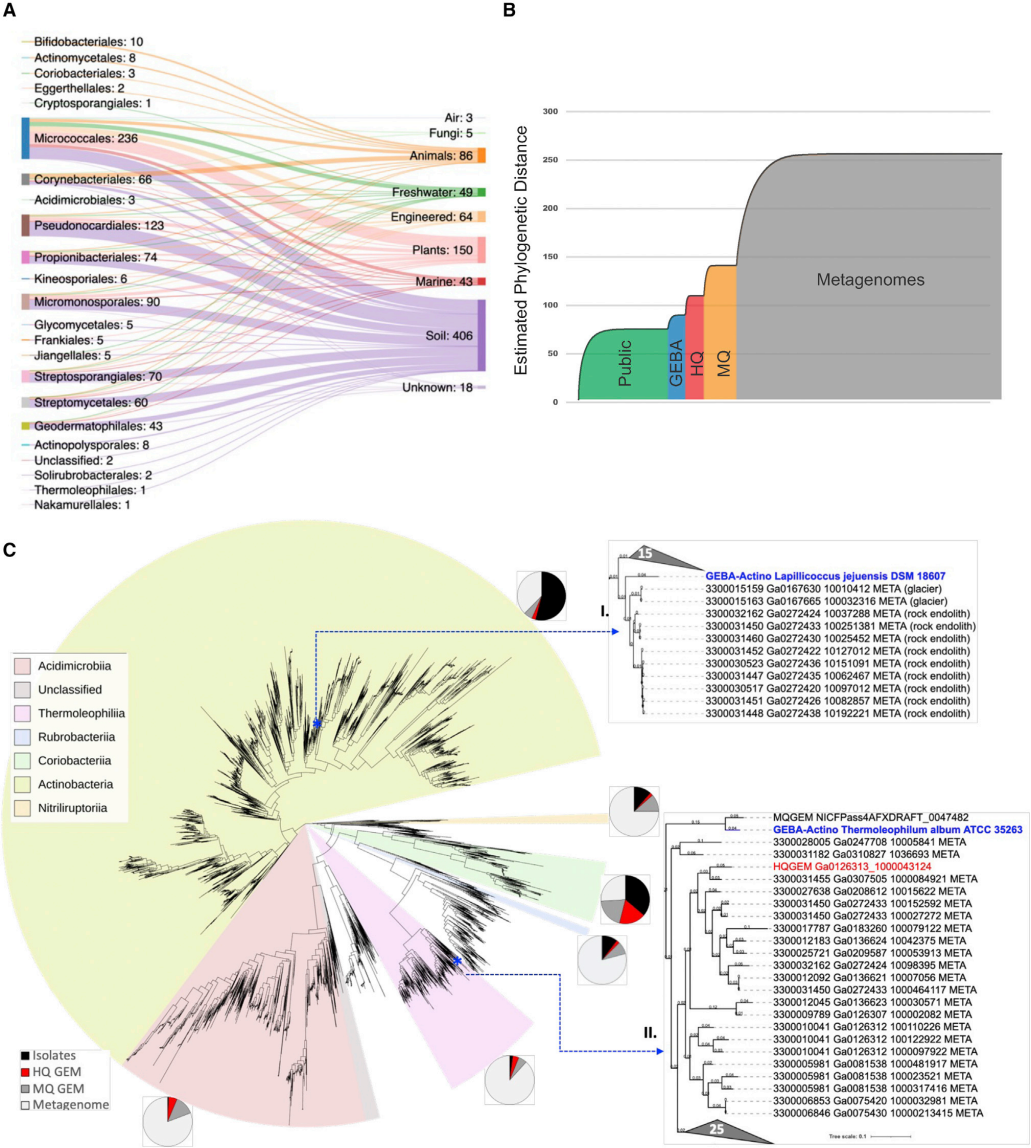
Phylogenetic diversity (PD) of phylum *Actinobacteria* (A) A total of 824 isolate genomes were sequenced from diverse taxa and habitats. Snapshot of taxonomic (order level) composition and isolation source of the 824 GEBA-Actino genomes is presented. Number of genomes attributed to each taxon or isolation source is shown next to each label. (B) PD accumulation curve depicting incremental increase in PD inferred from computed branch lengths of RpoB tree. The units on the x axis represent individual taxa or their equivalents (arising from metagenomes) ordered by genome category as the “accumulation units”: isolates (Public in green and GEBA in blue), MAGs (HQ in red and MQ in orange), and metagenomic sequences in gray. PD score based on summed branch lengths is shown on the y axis. (C) RpoB gene-based maximum likelihood phylogenetic tree used for PD calculation. The tree was rooted based on a representative set of archaeal RpoB sequences. For visualization purposes, clades with zero branch lengths were collapsed, and a single clade representative was retained. Individual actinobacterial classes are colored as indicated using the iToL interface.^18^ Uncolored sectors indicate operational taxonomic units (OTUs) composed entirely of uncultivated (metagenome and MAG) signatures. Pie charts indicate the proportion of isolate versus uncultivated sequences contributing leaves to each designated class. Inset trees show clades within class *Actinobacteria* (inset I) or class *Thermoleophilia* (inset II), highlighting GEBA type strains that could inform cultivation of members of adjoining uncultivated clades.

The investigated GEBA-Actino genomes represent 230 genera (54 families, 24 orders) from 4 classes: *Actinobacteria*, *Coriobacteriia*, *Acidimicrobiia*, and *Thermoleophilia*. Compared with other classes, which may be somewhat niche restricted, the class *Actinobacteria* is the largest and most diverse. The dataset includes the first sequenced representatives of 81 genera, expanding diversity in three unrepresented families (*Thermoleophilaceae*, *Rarobacteraceae*, *Motilibacteraceae*) as well as unclassified ones. *Thermoleophilum album* is the first sequenced isolate of the order *Thermoleophilales*. The overall taxonomic composition and isolation sources of the GEBA-Actino genomes are shown in Figure 1A and Table S1. GEBA-Actino type strains originate mainly from terrestrial and plant-associated habitats (Figure 1A), including some from extreme or unusual environments (e.g., alkaline, arid, permafrost, hypersaline, deep marine sediment) and non-human hosts such as sponges, fungi, and insects. These non-model microbes from environments posing unique metabolic challenges are of particular interest for the discovery of novel secondary metabolite prospects such as those with low toxicity to animals^1^^,^^15^^,^^16^^,^^17^ and also enable inquiry into habitat-specific adaptations through comparative genomics.

For comparative analysis purposes, a dereplicated set of 4,824 publicly available isolate genomes (referred to as “Public”) and 1,098 metagenome-assembled genomes (MAGs) from the comprehensive genomic catalog of Earth’s microbiomes^19^ was included (see STAR Methods and additional worksheets in Table S1). MAGs contributed significantly to the diversity of taxa, especially for classes underrepresented by isolates (*Coriobacteriia*, *Acidimicrobiia*, *Thermoleophilia*) (Figure S1). Notably, MAGs have 2.7 Mbp avg. genome size compared with 5.48 Mbp for isolates (Figure S2A). While this may be a potential bias due to lower completeness of MAGs or the difficulty of assembling larger genomes from metagenomics data, it may also reflect biases in phylogenetic and sample habitat composition and speak to reasons for their relative un-cultivability. MAGs also tend to be more fragmented with avg. scaffold length N50 of 131 Kbp (for MAGs) compared with over 1.88 Mbp avg. for GEBA-Actino (or 1.4 Mbp for all isolates). These differences are highlighted here since they impact downstream choices for analytical methods as well as results and biological inferences (Figure S2). More importantly, they emphasize the unique value of isolate genome sequences, particularly in the case of large and complex genomes of *Actinobacteria*.

### Status of the “uncultivated iceberg” for *Actinobacteria*

The “great plate count anomaly experiment”^20^ revealed that the vast majority (>99%) of microbial lineages were uncultivated and, consequently, unstudied. This concept is frequently illustrated by the disproportionately larger mass of submerged ice in the metaphorical iceberg. Given the multitude of recently sequenced genomes from both cultivated and uncultivated sources (due to innovations in metagenome assembly and binning methodologies), we revisited this precept as it pertains to members of the phylum *Actinobacteria*. We estimated the phylogenetic diversity (PD) of actinobacterial taxa, a simple and effective measure of biodiversity based on summing the branch lengths connecting those taxa on a phylogenetic tree.^21^^,^^22^ The maximum likelihood tree was generated based on universal single-copy marker genes identified from 5,648 isolate genomes (GEBA-Actino and Public), 3,321 MAGs (high quality [HQ] plus medium quality [MQ]), and over 20,000 metagenomes from diverse environmental samples (see STAR Methods). This analysis revealed that *Actinobacteria* isolate genomes account for only 34.68% of the total estimated diversity of the phylum (Figure 1B). While the contribution of HQ MAGs is relatively minor, including MQ MAGs boosts the coverage to 54.72% of total PD. This leaves close to 50% of actinobacterial diversity without any genome representation, highlighting the difficulty of genome recovery from metagenomics datasets. At the class level, isolates account for 60.25% of total PD of class *Actinobacteria* (Figure S3A), the largest and most diverse class within the phylum, and to which most isolates belong (Figure S1). There is a negligible boost from HQ MAGs, again pointing to possible difficulties in recovering such MAGs for large and complex actinobacterial genomes. For class *Coriobacteriia*, >45.31% is captured by isolates, while HQ MAGs boost coverage to well over 83.55% (Figure S3B) of this primarily host-associated taxonomic group with smaller genomes (Figure S2C).

Several clades of *Actinobacteria* were almost exclusively represented by metagenomic signatures or MAGs (Figure 1C). An examination of a sample source of these enigmatic clades reveals that new diversity arises from aquatic and terrestrial environments and notably, extreme, or nutrient-limited environments like sulfur acidic soils, peat permafrost, rocks, polar desert, and uranium-contaminated soils (Table S2). These clades include divergent members of classes with few to no isolate representatives (e.g., *Acidimicrobiia*, *Thermoleophilia*, *Rubrobacteriia*), as well as potentially new unclassified taxonomic groups (Figure 1C). Targeting extreme or nutrient-limited environments using standard or high-throughput cultivation strategies may result in the capture of these unrepresented lineages.^23^ Where related, GEBA type strains can help guide cultivation of specific uncultivated subclades (Figure 1C, insets) since their phenotypic, growth, and other requirements are well documented within curated databases like BacDive.^11^ For example, *Lapillicoccus jejuensis* DSM 18607, a well-characterized stone isolate,^24^ may serve as an appropriate reference for an uncultivated clade of rock-dwelling endoliths within the family *Intrasporangiaceae* (Figure 1C, inset I).

### Adaptations to the host or other environment

**Figure 2:**
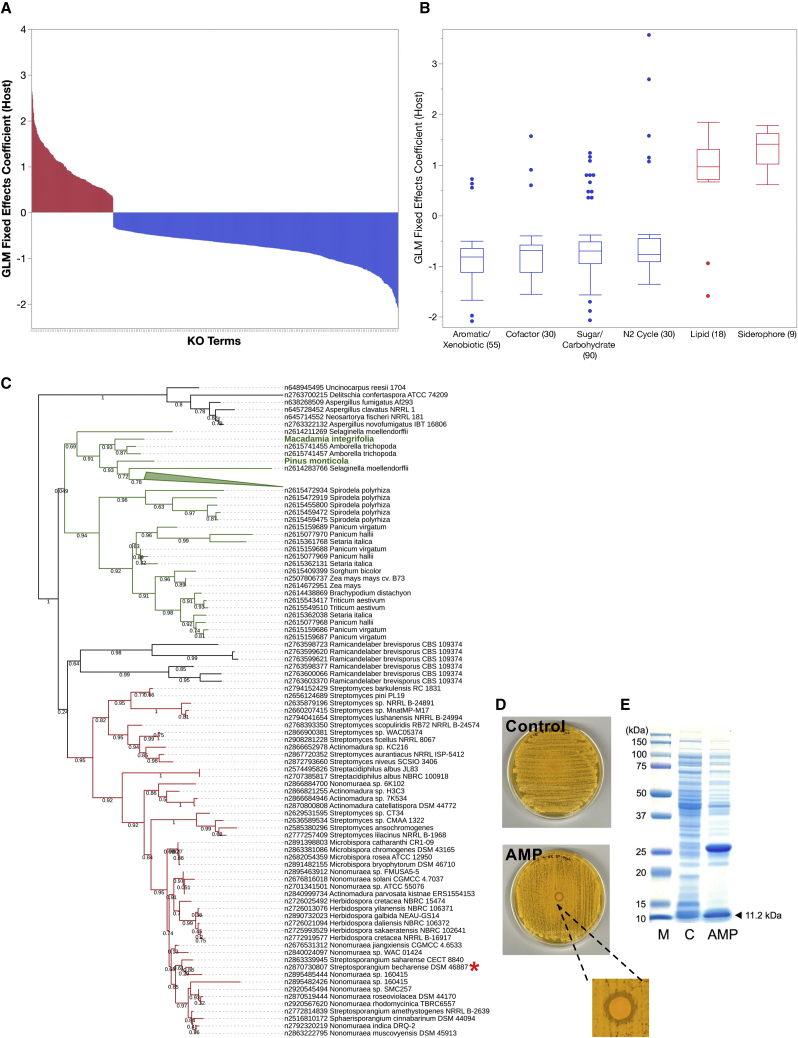
Functional adaptations of host versus environmental *Actinobacteria* (A) Significantly over- or underrepresented functions (KO terms, FDR-adjusted [adj.] p < 0.005) in host-associated versus other environmental genomes are shown. The x axis shows individual KO terms, while the y axis shows the logistic regression coefficient from a fixed-effect generalized linear model. Positive values (in red) indicate overrepresentation in host-associated genomes, while negative values (in blue) indicate overrepresentation in environmental group genomes. (B) Distribution of logistic regression coefficients (y axis) for individual KO function categories (x axis, discussed in the main text) is shown. Number of individual KO terms within each function category is shown in parentheses. Blue boxplots denote categories that are overrepresented in the environmental group, while red boxes denote categories in the host-associated group. (C) Maximum likelihood tree of eukaryal and bacterial candidate sequences assigned to PF09117. Characterized plant reference sequences are highlighted with green text. Bacterial branches are colored red, plant branches are green, and fungal branches are black. (D) Inhibition of *Saccharomyces cerevisiae* by AMP candidate of *Streptosporangium becharense* DSM 46887 overexpressed in *E. coli*. (E) SDS-PAGE gel showing the overexpression of recombinant AMP in *E. coli*. Lanes are protein size marker (M), control strain (C), and AMP-producing strain (AMP), respectively. The expected 11.2 kDa band of the AMP is highlighted.

We compared genomes of host-associated (2,650 genomes including 678 MAGs) versus environmental (2,306 including 284 MAGs) organisms to identify novel pathways or factors that may be attributed to adaptation to different lifestyles. Using a phylogeny-normalized generalized linear model approach, we identified protein families (Pfams, or KEGG Orthology [KO] terms) that were overrepresented in host-associated or environmental groups (Table S3). For example, out of 6,546 KO terms captured by 4,956 genomes, 1,100 were significantly (false discovery rate [FDR]-adjusted p < 0.005) overrepresented in either group (Figure 2A). Environmental genomes were notably enriched in functions related to the degradation of various aromatic or xenobiotic compounds, uptake and utilization of sugars, and carbohydrate-active enzymes (CAZYmes) for degradation of plant lignocellulose (e.g., cellulose, hemicellulose, pectin) (Figure 2B). These results could largely be attributed to many soil-dwelling terrestrial isolates in this group (Figure S1B). Similar observations were made using Pfams (Table S3). Other overrepresented functions include nitrogen cycling, cofactor biosynthesis, various transporters and regulators, and, interestingly, known determinants of plant growth promotion like pyrroloquinoline (PQQ) synthesis,^25^ 1-aminocyclopropane-1-carboxylate deaminase (ACCase),^26^ and phytase.^27^

Conversely, about 238 KO terms were overrepresented in the host-associated group—this relatively smaller number of enriched KO terms may reflect the smaller genome sizes (and consequently smaller functional repertoire) of host-associated genomes (Figure S2E). Among the enriched functions were known determinants of pathogenesis or host interaction like adhesins, siderophores, lactocepin, lysozyme inhibitor, and steroid degradation enzymes.^28^^,^^29^^,^^30^ Additionally, we found several potential markers of adaptation to anaerobic conditions, including the FeoABC system for ferrous iron uptake, anaerobic ribonucleoside-triphosphate reductase, and C4-dicarboxylate membrane transporter.^31^ More than 15 KO terms for lipid metabolism are noteworthy (Figure 2B) and may play a role in host-derived fatty acid utilization—e.g., fatty acid coenzyme A (CoA) ligases (K12421, K12422, K12423, K12427, K12428, K01909), acyltransferases, acyl-coA synthetase, and others^32^^,^^33^ (Table S3).

Other significantly over- or underrepresented functions are potentially less well understood or characterized in bacteria—for example, Pfams with limited phylogenetic distribution (LPD) or potential eukaryal origin within the host-overrepresented set are demarcated based on proportions of sequences recruited to individual Pfams from the 100,000+ isolate genomes of bacteria, archaea, or eukarya stored in the IMG database. For example, an arthropod defensin (PF01097, 91% eukaryal candidate sequences) from insects and scorpions with activity against Gram-positive bacterial pathogens may be similarly employed by members of *Actinomyce*s spp.^34^ The roles of other eukaryal-like Pfams may be more cryptic, like PF01490 (amino acid transporter, 94% eukaryal) found in *Corynebacterium* spp. and *Kocuria* spp., or PF05241 (expanded emopamil binding protein superfamily including characterized sterol isomerases, 84% eukaryal), which is restricted to several species of host-associated *Mycobacterium* spp., *Mycolicibacterium* spp., *Microbacterium* spp., and *Nocardia* spp., and are membrane bound (6 transmembrane regions on average). (Figure S4). A eukaryal phospholipase B (PF04916, 45% eukaryal) has remote homologs in *Bifidobacterium* spp., *Mycobacterium s*pp., and *Adlercreutzia* spp.; horizontal gene transfer among members residing in a shared niche is conjectured (e.g., between *Bifidobacterium* sp. and *Lactobacillus* sp.) (Figure S5).

A potential novel antimicrobial peptide or AMP (PF09117, 96% eukaryal) is detected only in a small subset of soil- and plant-associated *Actinobacteria* outside of plant and fungal genomes (Figure 2C). We demonstrate inhibition of *Saccharomyces cerevisiae* by an AMP candidate from *Streptosporangium becharense* DSM 46887 cloned into *E. coli* (see STAR Methods; Figure 2D). A potential dimeric form of the AMP is suggested by the presence of a ∼25 kDa band in addition to the expected 11.2 kDa product on an SDS-PAGE gel (Figure 2E). AMP dimerism has been previously reported.^35^^,^^36^ The sequence lengths of 59 candidate actinobacterial AMPs varied from 101 to 121 amino acids with a median length of 102 residues. An N-terminal signal peptide was detected in every instance. A survey of gene neighborhoods revealed no conserved colocalized functions. AMPs are a promising new class of therapeutic antibiotics displaying broad-spectrum antimicrobial efficacy against bacteria, fungi, and viruses.^37^^,^^38^^,^^39^

LPD Pfams showing a discordant phylogenetic distribution within a narrow subset of bacterial lineages are also intriguing—e.g., DUF4300 (PF14133) was detected in known pathogenic or host-associated lineages within *Actinobacteria* and a few other bacteria phyla (Figure S4). This and other examples are described in Data S1. Many other comparisons are possible depending on the availability of underlying metadata, highlighting interesting targets for experimental investigation. For example, notable differences arising from genome comparisons of plant (195) versus animal (214) host isolates of the order *Micrococcales* include the uptake and utilization of known plant sugars like rhamnose or xylose and the utilization of GABA (a plant signal), ACCase (a well-recognized plant-growth-promoting factor), flagellar components, urate catabolism, etc. Similarly, for animal-associated isolates, enrichment of known virulence determinants like autotransporters and adhesins were found along with markers of anaerobiosis, antibiotic resistance, toxin/antitoxin systems, CRISPR-Cas systems, and many LPD families (Table S4).

### Shaping of the secondary metabolite repertoire

*Actinobacteria* have been the focus of natural product or secondary metabolites (SMs) discovery for decades, and large-scale genomics has illuminated thousands of BGCs with the potential for new therapeutic and antimicrobial applications.^4^^,^^40^^,^^41^^,^^42^^,^^43^ Beyond defense and competition, SMs can mediate diverse biotic interactions (including cooperative ones) like communication, nutrient acquisition, metal scavenging, stress protection, phage induction, and more, all of which can influence microbial fitness with impacts on microbial ecology and evolution. Here, we analyzed BGCs for SM production across all 5,648 isolate genomes using AntiSMASH 6.^44^ A total of 80,947 BGCs were predicted from 5,194 genomes (out of 5,648) (Table S5; Data S2). These were assigned to 44,923 distinct gene cluster families (GCFs) using BiG-SLICE (Table S6), of which 32,570 were singletons, while the largest-sized GCFs with >100 BGCs included non-ribosomal peptide synthases (NRPSs) (1,040 BGCs), siderophores (523), RiPP-like (297), ectoine (259), terpene (193), etc. (Figure S6). The taxonomic composition of most of these GCFs was broad with a few exceptions like a siderophore (GCF ID 249228), RiPP-like (ID 249163), and terpene (ID 252912), restricted primarily to various *Streptomyces* spp., an ectoine (ID 251253) restricted to *Rhodococcus* spp., or a 98-member terpene GCF (ID 251612) from *Micromonospora* spp. A total of 6,939 GCFs were contributed exclusively by 744 GEBA-Actino genomes from the current study, 822 of which arise from 94 new genera. These results agree with the recent survey of BGCs by Gavriilidou et al. that highlight *Actinobacteria* (particularly *Streptomyces*, *Amycolatopsis*, *Kutzneria*, and *Micromonospora*) as top contributors of GCF diversity across all bacterial phyla.^43^

Overall, NRPS, terpenes, and type I polyketide synthase (T1PKS) were the most abundant SM classes, with terpenes (and, to a lesser extent, T3PKS, RiPP-like, and betalactones) widely distributed across genera. Other classes of SMs showed highly sporadic or phylogenetically incongruent distribution, alluding to widespread horizontal gene transfer of SMs, which is explored further below. Only 2,609 (3.2%) of the total BGCs had a significant (≥80% identity over ≥80% of the reference sequence) hit to the manually curated MIBiG BGCs of known function.^45^ At ≥90% identity, a mere 1,155 (1.4%) had hits, a low value similar to those reported in other studies,^19^ since the vast majority of BGC products have not been chemically characterized or otherwise experimentally validated.

**Figure 3:**
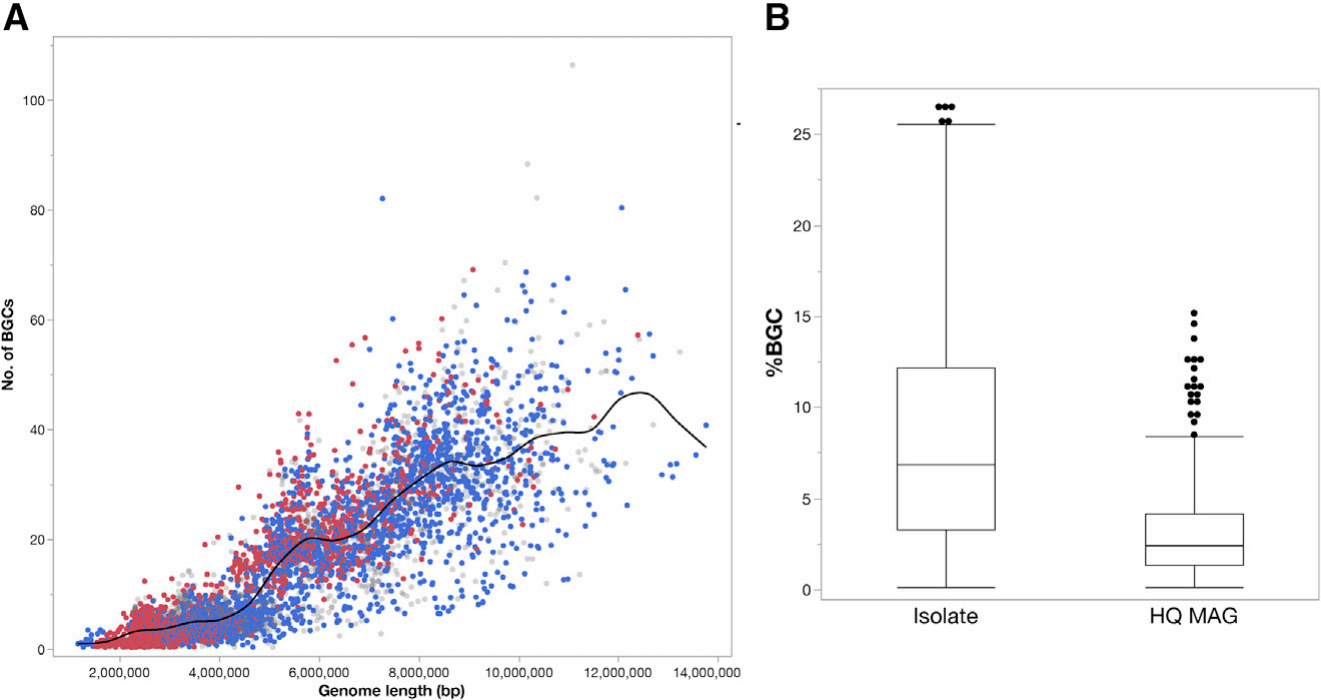
Overview of BGC abundances across actinobacterial genomes (A) Relationship between genome size and total number of predicted BGCs per genome. Data points are colored based on isolation source (where available). The x axis is the genome size (in Mbp), and the y axis is the total number of BGCs. (B) Distribution of percentage of BGCs (total BGC length as percentage of total genome length) for isolate genomes (including GEBA and public) compared with HQ MAGs.

As expected, there was a positive trend between genome size and the number of BGCs^46^^,^^47^ with an avg. of 15.58 BGCs detected per genome, comprising 8.05% of total genome length, hereafter referred to as %BGCs (Figures 3A and 3B). Host-associated genome sizes were smaller on avg. than environmental genomes (Figure 1B) and encoded fewer BGCs, which comprised, on avg., 7.15% BGCs compared with 9.09% BGCs for environmental genomes (Figure S7). *Kitasatospora kifunensis* DSM 41654, a soil isolate, displayed the top BGC commitment with 26.50% BGCs (Table S5). Other genomes with notable BGC commitment included several *Streptomyces* spp., *Nocardia* spp., and newly sequenced genera from the GEBA-Actino set (e.g., *Goodfellowiella coeruleoviolacea* DSM 43935, *Actinocrispum wychmicini* DSM 45934, *Labedaea rhizosphaerae* DSM 45361). BGC commitment is summarized by various taxonomic levels in Figures S8A–S8C.

No BGCs could be predicted in 454 isolate genomes using AntiSMASH or an alternate machine-learning-based method, DeepBGC.^48^ These were almost entirely small host-associated genomes (2.2 Mbp median length; Figure S9). The few exceptions included genomes of terrestrial *Nocardioides* spp. with genome size up to 5 Mbp (avg. > 99% completeness)—other *Nocardioides* spp. (from diverse environments) showed very low BGC commitment (avg. 2.63% BGCs). An inspection of individual genera that contained species both with and without BGCs demonstrated consistent patterns of BGC presence or absence in individual subclades—for example, the relative loss of the solitary type III polyketide synthase cluster in the last common ancestor of a subclade of *Gardnerella vaginalis* strains (Figure S10). A discontinuous distribution of individual SM classes in *Bifidobacterium* species again suggests relative gains and losses (Figure S11A)—for example, an interrupted pattern of lanthipeptide in *B. pseudocatenulatum* DSM 20438 and DC2A can be attributed to inactivation by truncation or point mutation of a lanthipeptide “hook” protein (Figure S11B), while no marker genes are detectable in strain L15. A phenazine-like BGC (containing PhzA/B but no other genes associated with a canonical phenazine operon)^49^ detected in all strains of a discrete *B. thermophilum* clade, but with few instances elsewhere in the genus, suggest potential acquisition by a last common ancestor of this cohort (Figure S11A).

This pattern of sporadic distribution of SM type is the rule, rather than the exception, and is observed within every genus and most species, echoing individual reports.^50^^,^^51^^,^^52^ The hypothesis follows that horizontal gene transfer (HGT) drives expansion of SM repertoires due to variable evolutionary pressures, even for narrow sublineages. To address this, we cross-referenced BGC-containing scaffolds with a list of scaffolds designated as putative plasmids by at least two independent prediction methods. A total of 936 plasmids bearing one or more BGC (1,119 total) were identified from 659 genomes belonging to 74 genera (11,999 plasmid scaffolds from 2,920 genomes from 240 genera were predicted with or without a BGC, and these are presented in Table S7). The length of BGC-encoding plasmids ranged from 2,535 (partial plasmid lengths due to higher fragmentation of some draft genomes is possible) up to 1,356,931 bp (Figure S12). All megaplasmids (>500 Kbp) are detected in terrestrial isolates, and some have been previously reported.^53^^,^^54^

Examining numbers of genomes with BGC-bearing plasmids at the genus level, *Streptomyces* spp., *Rhodococcus* spp., *Frankia* spp., and *Salinispora* spp. are examples employing plasmids as a prominent strategy for BGC expansion, in addition to *Pseudonocardia* spp., *Actinomadura* spp., *Mycobacterium* spp. etc. (Figure S13; Table S5). The role of plasmids in shaping the SM repertoire of a small subset of *Streptomyces* spp. and *Rhodococcus* spp. have been previously examined.^53^^,^^55^ The contribution of plasmid-borne BGCs to the total number or total %BGCs per genome ranges from <1% to 66.6% (three genomes have a solitary BGC that is located on a plasmid). While there was no clear preponderance of plasmid-encoded SM classes, lanthipeptide-class-I, thioamitides, and butyrolactone were relatively overrepresented. Genera like *Mycobacteroides*, *Rathayibacter*, *Gordonia*, *Mycolicibacterium*, and others have above avg. %BGC commitment but do not appear to employ plasmids for BGC expansion.

**Figure 4:**
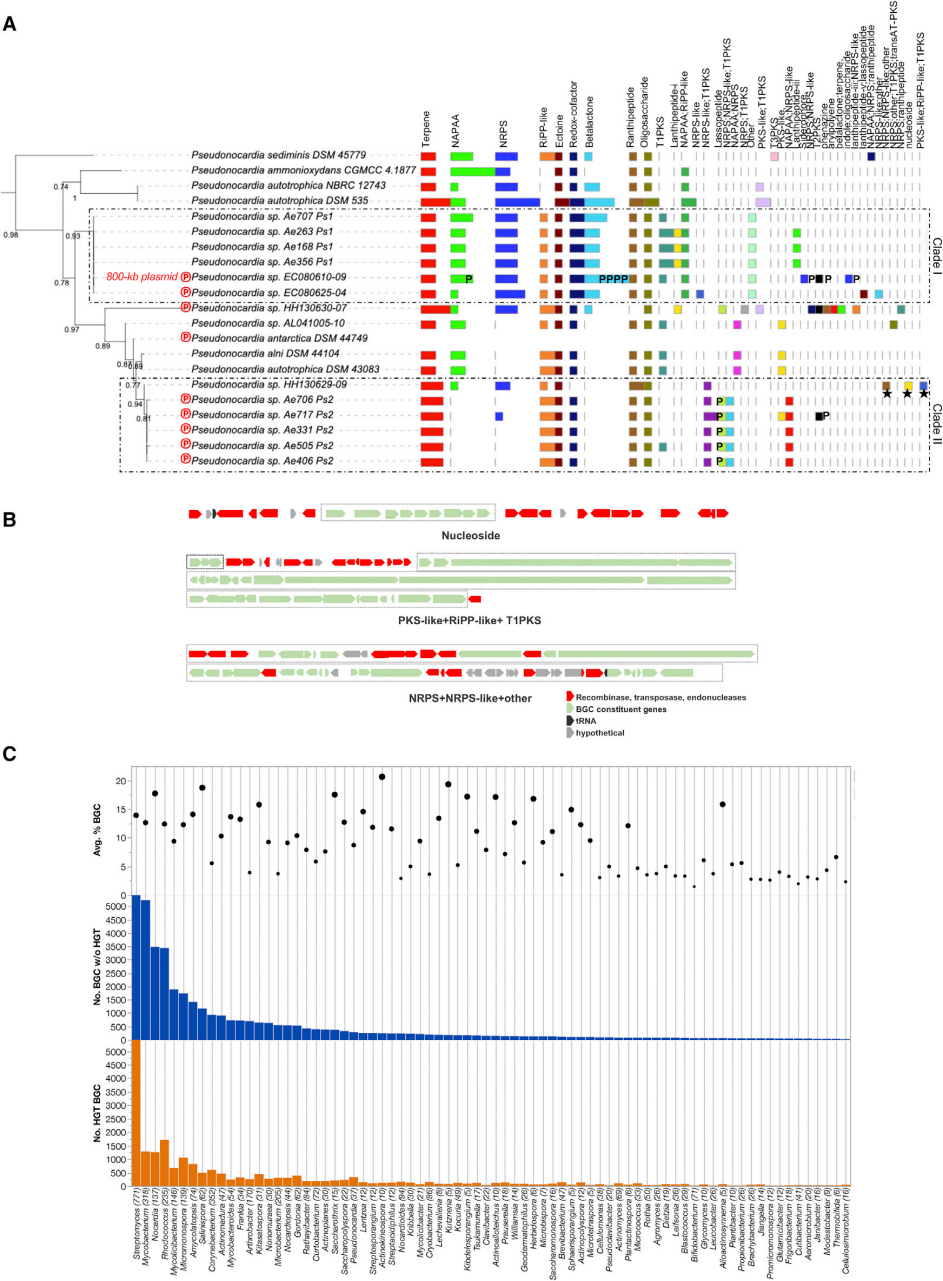
Horizontal gene transfer of BGCs (A) Examining the role of plasmid-mediated HGT within a closely related subset of *Pseudonocardia* spp. Maximum likelihood tree based on RpoB gene alignments of selected strains is annotated with bar charts depicting the number of BGCs for each class predicted by AntiSMASH (drawn using iToL). Bootstrap support is included. “P” is used to indicate a BGC detected on a plasmid scaffold predicted in that genome. “℗” adjacent to a genome label indicates a BGC-bearing plasmid. Other genomes may have plasmids, but BGCs were not encoded on those plasmids. Subclades are highlighted as discussed in the manuscript. Black stars mark further instances of HGT as illustrated in (B). (B) Schematic of BGC examples in strain HH130629-09 that may have been acquired via alternative means of HGT such as ICEs. The core genes for the BGC are colored green, while red indicates hallmark genes for integration or transposition. tRNA genes are shown in black. (C) Genera encoding the highest numbers of HGT BGCs (orange bars) are contrasted with non-HGT BGCs (blue bars). Bars for *Streptomyces* are truncated for better display and total 11,018 HGT BGCs versus 15,507 non-HGT BGCs. Top panel with weighted points is the average percentage of BGCs of genomes in each genus. On the x axis, genera are ordered by descending order of total number of BGCs without HGT. Number of genomes for each genus is shown in parentheses.

In *Pseudonocardia* spp., multiple plasmid-borne BGCs are in evidence (Figure 4A)—for example, an 800 kb megaplasmid in strain EC080610-09 results in eight new strain-specific BGCs (see subclade I). In subclade II, all possess a lassopeptide-bearing plasmid (Figure 4A) except for a near-identical strain, HH130629-09, that is missing this (or any other) plasmid but possesses additional strain-specific SMs. Examining their gene neighborhoods reveals they are flanked by transposases, integrases, recombinases, etc., suggesting that other means of HGT may have been employed (Figure 4B). BGCs for nucleoside and NRPS + other appear to be inserted at tRNA genes, suggesting they may have been borne on integrative and mobilizable or conjugative elements (IMEs or ICEs, respectively).^56^^,^^57^

To survey this more systematically across the entire dataset, we cross-referenced BGCs against HGT-derived genes predicted by HGTector^58^ and found that 28,913 BGCs from 4,776 genomes have predicted HGT genes. 457 of these BGCs were found on predicted plasmids. This implies that most genomes may possess at least one horizontally acquired BGC. The proportion of such HGT BGCs ranges from 2.85% to 100% (median 38%) of the total number of BGCs per genome. 178 genomes showed 100% HGT rate; however, most of these belonged to small host-associated genera with a single BGC—such as several species of *Actinomyces*, *Bifidobacterium*, *Cutibacterium*, *Candidatus Planktophila*, etc. Other genera with a striking proportion of HGT-derived BGCs as well as high BGC commitment included *Streptomyces* (41.5% of total BGCs from 770 genomes), *Rhodococcus* (33% from 235 genomes), *Micromonospora* (38% from 139 genomes), *Kitasatospora* (41% from 31 genomes), *Gordonia* (42% from 61 genomes), *Pseudonocardia* (54% from 37 genomes), etc. (Figure 4C). In *Streptomyces* spp., BGC flux mediated by plasmids or actinomycete IMEs and ICEs has previously been recognized.^59^

SM classes that are notably overrepresented in this HGT subset include terpene, RiPP-like, siderophore, ectoine, butyrolactone, redox-cofactor, melanin, etc. (Figure S14). A total of 313 out of 646 *Pseudonocardia* spp. BGCs appear in this list, including the strain-specific ones highlighted above. Similarly, in *Bifidobacterium* spp., 23 BGCs in 21 genomes may have been recently acquired (Figure S11A). Overall, subclades within each lineage are likely under different ongoing selective pressures driving the highly dissimilar BGC composition, facilitated by various HGT strategies as well as deletion events.^60^ This evidence of relatively recent acquisition may be used as a strategy for prioritizing characterization of specific BGCs in addition to previously suggested ones.^61^

Horizontal transfer may impact the detection of BGCs even in HQ MAGs—for example, MAGs encoded only 3 BGCs/genome or 2.39% BGCs (Figure 3B). These are possible underpredictions since the metagenome-binning process is expected to be biased against HGT regions due to their deviant nucleotide composition and/or coverage (plasmid copy-number effects), compared with the main chromosome.^62^ Furthermore, the higher relative fragmentation of MAGs (avg. scaffold N50 of 141.6 Kbp for HQ MAGs versus 1.4 Mbp for all isolates) can also contribute to false negatives since BGC lengths avg. > 33 kb (based on MiBIG^45^ and GenBank entries). This further underscores the need for HQ isolate genome sequences for continued SM gene discovery efforts.^63^^,^^64^

### Prophages and host-virus interactions

Prophages are phage genomes residing in bacterial cells, often integrated into their host chromosome, during latent phases of their infection cycles. In addition to contributing to HGT, phage-host interactions may also play a role in iterative genome evolution and possibly contribute to host fitness by conferring resistance mechanisms or metabolic advantages. Identifying prophages from whole-genome data provides a unique opportunity to better understand the prevalence, diversity, host range, and gene content of phages infecting *Actinobacteria*.

We applied VirSorter2^65^ and CheckV^66^ to automatically detect, curate, and identify (near-)complete prophage sequences in *Actinobacteria* isolate genomes (see STAR Methods; Figure S15). After quality filtering and dereplication, a final dataset of 4,831 distinct prophages from 2,756 genomes was obtained, including 3,393 estimated to be (near-)complete from 2,244 genomes. We then mapped predicted proteins from all *Actinobacteria* isolate genomes to this non-redundant catalog of *Actinobacteria* prophages to establish a global picture of prophage prevalence and distribution across *Actinobacteria*.

**Figure 5:**
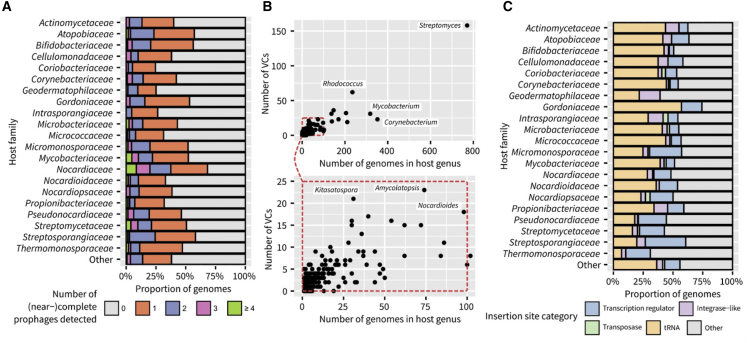
Overview of prophage content across *Actinobacteria* genomes (A) Number of complete and near-complete prophages detected by genomes across major families. Families with ≤50 genomes are gathered in the “other” category. (B) Number of distinct viral clusters detected by host genus, relative to the number of genomes screened in the genus. The bottom panel shows a zoomed-in version of the data for genera with ≤105 genomes. Individual genera with the most VCs and/or genomes mined are named on each plot. (C) Prophage insertion site across major *Actinobacteria* families. For each prophage, the host genomic regions immediately 1 kb upstream and downstream of the 5′ and 3′ ends, respectively, were screened for the detection of tRNA, integrase-like genes, or transposases belonging to other mobile genetic elements (i.e., not the prophage currently considered), and transcription regulators. Families with ≤50 genomes are gathered in the “other” category.

Overall, 60.4% of *Actinobacteria* isolate genomes (n = 3,412) included at least one prophage-like region (Table S8), while a complete or near-complete prophage could be detected in 45.4% of the genomes. This difference is likely due to the presence of inactive and/or decayed prophages and to challenges in assembling variable genome regions, including prophages, from short reads. The relatively high frequency of genomes without any detectable prophages across *Actinobacteria* (∼40%) is in line with previous observations^67^^,^^68^^,^^69^ and seems to be consistent across taxa within the *Actinobacteria* phylum (Figure 5A). Overall, the number of prophages detected per genus scaled with the number of genomes sequenced within this genus (Pearson correlation coefficient = 0.89) with a handful of outliers. First, strains in the *Mycobacteroides*, *Bifidobacterium*, and *Leifsonia* yielded a disproportionately large number of prophages and consistently displayed a lower percentage of genomes without any trace of prophage compared with other genera (19%, 26%, and 31% respectively). In the case of *Mycobacteroides*, this may be due in part to the large collection of phages isolated from strains in this genus,^70^ which may help with the identification of (HQ) prophages. On the other end of the spectrum, *Clavibacter* strains included 90% of genomes without any trace of a prophage. Since *Clavibacter* genomes are relatively compact (∼3 Mb), they may include less prophages than other larger *Actinobacteria* genomes; however, it is also possible that *Clavibacter* prophages are simply more distant from references and more challenging to detect than other *Actinobacteria* phages.

When present however, it is not infrequent to observe multiple distinct prophages in the same host genome (17% overall), which could provide opportunities for recombination and HGT between unrelated phages. This is consistent with some temperate actinophages having been identified as exhibiting “high lateral gene flow” pattern, i.e., subject to a higher rate of horizontal gene exchange than most other phages.^71^ Among various genome features including genome size, isolation source, host taxonomy at the genus, family, or order rank, number of tRNAs, and presence of CRISPR-CAS systems and BGCs, only taxonomy was detected as significantly associated with the number of prophages detected (ANOVA p value < 2 × 10^−16^ at all ranks tested). This indicates that the variation in prophage presence is not directly linked to a general environment or lifestyle (e.g., SM producer) but instead is likely due to differences in life-history traits between strains, which are likely best captured as taxonomic classification in this dataset.^72^

Next, we evaluated the diversity of prophages recovered across *Actinobacteria* genomes through automated phage genome network analysis implemented in vContact 2.^73^ Clustering all (near-)complete *Actinobacteria* prophages along with 14,256 reference genomes from the INPHARED database^74^ yielded a total of ∼1,837 genus-level groups (i.e., viral clusters [VCs]), including 365 with ≥2 phages. Almost half (46%) of host genera were associated with 2 or more VCs, and the number of VCs detected per genus was clearly increasing with the number of genomes sampled in the host genus (Figure 5B). This illustrates how *Actinobacteria* within individual genera can be infected by a broad range of phages and how whole-genome shotgun sequencing of many members within a given genus can shed light on this extensive prophage diversity.

Given this broad phage diversity, we next evaluated the distribution of individual prophages across host diversity. Prophages were typically (78%) detected in a single genome and, when detected in multiple genomes, were majorly associated with a single genus (85%; Figure S16). When detected across multiple genera, however, the host genera tended to be in different families (58%) and order (45%; Figure S16). This suggests that while most *Actinobacteria* prophages are “specialists,” i.e., have a narrow host range, the host range of “generalist” prophages does not closely reflect host taxonomy beyond the genus rank. Conversely, individual VCs were much more frequently detected across diverse hosts (Figure S16). Among VCs with 2 or more prophages, 50% were associated with more than one host genus and 25% with multiple host families. Several VCs also included members infecting multiple classes of *Actinobacteria*, suggesting that these either reflected ancient groups of phages predating the divergence of these different classes or, more likely, that some prophages were able to “jump” from one host to another in a different class.

Finally, we explored the gene content of *Actinobacteria* prophages to evaluate the potential impact of prophage on host cell functioning. As is typical in phage genomes, most genes (60%–80% depending on the host genus) could not be functionally annotated, while the annotated functions were mostly directly related to phage replication and capsid production, e.g., integrases, major capsid proteins, or tail proteins. However, one exception, a gene encoding a component of predicted Mn/Zn uptake complex, was identified in 3 *Atopobium* prophages (Figure S17). Zn uptake can play a critical role in the pathogenicity of some bacteria,^75^ and the presence of phage-encoded Mn/Zn transporters suggests that some *Actinobacteria* prophages may directly increase their host’s fitness by providing additional resources for acquiring these nutrients. Beyond phage-encoded genes, however, prophage integration can also influence host cell functioning by disrupting neighboring genes.

Since the vast majority (94.7%) of *Actinobacteria* prophages were detected as integrated in the host chromosome, we also explored the function of genes found near insertion sites. For 65% of integrated prophages, an integrase-like and/or tRNA gene could be identified on the phage side of the integration sites at the 5′ or 3′ end, as is typical for *Caudoviricetes* (Table S8). In contrast, the genes found immediately outside of prophage insertion sites were much more variable (Figure 5C). Notably, these included a substantial number of transposases and integrases, distinct from the ones identified within the prophage and often belonging to other mobile genetic elements integrated immediately upstream/downstream of the prophage. This suggests that a number of *Actinobacteria* prophages may be integrated in integration hotspots, likely representing hypervariable regions of the *Actinobacteria* genome. The other common functional category identified immediately next to insertion sites was transcriptional regulators, suggesting that some prophage integration events may impact regulatory pathways within the host cell.^76^

### Conclusions

Microbial genomics has come a long way since the first bacterial whole-genome sequence of *Haemophilus influenzae* published in 1995^77^^,^^78^—as of March 2021, over 220,700 genomes of bacteria and archaea are listed in RefSeq.^79^ These numbers are of course dwarfed by those of uncultivated genome equivalents (MAGs and single-amplified genomes [SAGs]) derived from environmental samples. Many of these genomes from “dark matter” lineages like the candidate phylum radiation (CPR) and others upend microbial precepts arising from the study of experimentally tractable lineages and model organisms like *E. coli*.^80^^,^^81^^,^^82^^,^^83^ While this data deluge is impressive, the role and importance of HQ genomes of isolates is undeniable, not only in serving as a reference point for the interpretation of uncultivated sequences but also as an experimentally tractable resource in the laboratory.

Here, we explore *Actinobacteria*, a large and ancient phylum renowned for the richness and diversity of its natural products, by first producing HQ draft genomes of 824 isolates of primarily type strains. Comparative analyses with public genomes (both isolates and MAGs) revealed that only half of total actinobacterial PD is represented by a genome (even if including MQ MAGs with >50% incompleteness). A large portion of the remaining diversity can be attributed to underrepresented or new lineages arising from poorly accessible or extreme environments. Isolation efforts concentrated on such understudied or rare samples could result in the capture of a significant portion of this unrepresented diversity. The inherent value of well characterized type strains in informing cultivation of novel or unrepresented clades is also underscored with some examples.

The term “dark matter” may also be applied to the functionally unknown content within genomes (e.g., orphan genes, intergenic regions, proto-genes, etc.), which is even more extensive and intractable than the taxonomic dark matter. So, while the fraction of inaccessible taxa may diminish, the functional characterization lags far behind.^84^ Here, again, the value of type strains as accessible standardized material is obvious. With greater statistical power achievable due to increased numbers of genomes of *Actinobacteria* from diverse lineages and environments, robust genome-wide comparisons are feasible toward identifying adaptations specific to a lineage, environment, or observed genotype or phenotype. We identify new and uncharacterized functions involved in niche adaptation by comparing host-associated versus environmental genomes. For example, several enriched Pfams for lipid metabolism may represent new or overlooked determinants of host-microbe interaction and possibly virulence, even in well-studied human pathogens. Functions with restricted taxonomic distribution are highlighted, and a previously uncharacterized antimicrobial peptide family enriched in soil- and plant-associated *Actinobacteria* is preliminarily characterized*.* Much more is possible with this expanded set of genomes accounting for almost 80% of projected diversity for the class *Actinobacteria* (the largest class)—underpinnings of phenotypes such as sporulation, cell shape, multicellularity, DNA topology, etc., await discovery (the only constraint being the availability of reliable metadata).

We also analyze an inventory of >80,000 BGCs predicted from isolates and examine the widespread role of HGT in shaping the repertoire across taxa. The ubiquity of this phenomenon and the highly fragmented nature of HQ MAGs results in a potential bias in BGC discovery, again reiterating the need for reference isolate genome sequences. However, the sequence itself is merely a starting point, and unfortunately only an insignificant fraction of over a million BGCs have any confirmed bioactivity, so the need for targeted efforts is great. To this end, the evolutionary and ecological history of a BGC, such as recent HGT events and its distribution in different environments, could provide an additional line of reasoning in prioritization of BGCs for biochemical characterization. Overall, our findings emphasize the essential role- and unique value of reference isolate genomes and present a compelling case for the continued sequencing of extant strains of isolates.

### Limitations of the study

While we have emphasized the value of HQ genomes of cultured species as a reference point for various analyses and experimentation, the procurement of such actinobacterial cultures is non-trivial—a cultivation bias due to predicted slow growth rates is likely for many, as recently indicated for marine actinobacteria.^85^ *Actinobacteria* are also known to have very large and highly repetitive genomes, which prevents their recovery from metagenomes. Furthermore, while existing isolate genomes are a notable resource, almost a quarter of coding sequences (CDSs) elicit no functional annotation, and the vast majority are uncharacterized.

## STAR★Methods

### Key resources table

**Table:** 

REAGENT or RESOURCE	SOURCE	IDENTIFIER
**Bacterial and virus strains**		

*Escherichia. coli* BL21(DE3)	ThermoFisher Scientific	Cat#EC0114
*Saccharomyces cerevisiae* CEN.PK2	EUROSCARF Institute for Molecular Biosciences	54398

**Chemicals, peptides, and recombinant proteins**		

LB (Miller’s) broth	Growcells	Cat#MBLE-7030
Carbenicillin	Sigma-Aldrich	Cat#C1613
Isopropyl β-D-1-thiogalactopyranoside (IPTG)	Sigma-Aldrich	Cat#I6758
BugBuster® Protein Extraction Reagent	Millpore	Cat#70584
YPD Agar Plates	TEKNOVA	Cat#Y5131
Fluconazole	Cerilliant®	Cat#F-097
Coomassie Brilliant Blue R-250 Staining Solution	Bio-Rad	Cat#1610436

**Deposited data**		

GEBA-Actino genomes generated by this study	This study	GenBank accessions provided in Table S1https://www.ncbi.nlm.nih.gov/genbank/
Metagenome-assembled genomes (MAGs) from GEM dataset	Nayfach et al.^19^	https://genome.jgi.doe.gov/GEMs

**Recombinant DNA**		

pET-21(+)_AMP_S.be: pET21-(+) plasmid containing AMP sequences originating from Streptosporangium becharense DSM 46887	This study	N/A

**Software and algorithms**		

Interactive tree of like (iTOL)	Letunic and Bork^18^	https://itol.embl.de/
PD estimation	This paper	https://doi.org/10.5281/zenodo.7058177
Generalized linear model analysis	This paper	https://doi.org/10.5281/zenodo.7058201

### Resource availability

#### Lead contact

Further information and requests for resources and analyses should be directed to Rekha Seshadri (rseshadri@lbl.gov).

#### Materials availability

This study did not generate new materials.

### Method details

#### Sequence, assembly and annotation

All GEBA-Actino genomes were sequenced at the DOE Joint Genome Institute (JGI) using Illumina technology^86^ or Pacific Biosciences (PacBio) RS technology.^87^ For all genomes, we either constructed and sequenced an Illumina short-insert paired-end library with an average insert size of 270 bp, or a Pacbio SMRTbell library. Genomes were assembled using Flye v. 2.6,^88^ ALLPATHS^89^ or Hierarchical Genome Assembly Process (HGAP)^90^ assembly methods (specifics provided in Table S1). Genomes were annotated by the DOE–JGI genome annotation pipeline.^91^ Briefly, protein-coding genes (CDSs) were identified using Prodigal^92^ followed by a round of automated and manual curation using the JGI GenePrimp pipeline.^93^ Functional annotation and additional analyses were performed within the Integrated Microbial Genomes (IMG-ER) platform.^14^ All GEBA-Actino data are available through the Integrated Microbial Genomes with Microbiomes (IMG/M) system^14^ and GenBank,^94^ and the corresponding type strains through the respective culture collections (Table S1). All data including detailed sequencing and assembly reports can be downloaded from GenBank and JGI Genome portal: https://genome.jgi.doe.gov/portal/

#### Curating public genomes for comparative analyses

For various comparisons described in the study, a total of 4,824 good quality phylum *Actinobacteria* isolate genomes were curated from the complete set of available public genomes (at the inception of this analysis in Jan 2020). “High quality” public genomes are designated by the IMG quality control pipeline (based on phylum-level taxonomic assignment or if the coding density is >70% or <100%, or the number of genes per million base pair is >300 or <1,200.^91^ CheckM completeness/contamination criteria were also applied with some exceptions such as highly reduced genomes of *Tropheryma* spp. (∼0.83 Mbp) that are likely underestimated by checkM due to loss of marker genes.^95^ Isolate genomes dataset was partially de-duplicated by removing multiple strains of *Mycobacterium tuberculosis, Mycobacteroides abscessus* (for example) after assessing the average nucleotide identity (ANI) of total best bidirectional hits and removing genomes sharing >99% ANI (alignment fraction of total CDS ≥ 90%) to another genome within that set. A total of 1,098 Actinobacterial MAGs were selected (based on ≥95% completeness and ≤5% contamination) from a recently published comprehensive catalog of MAGs recovered from over 10,000 public metagenomes representing the breadth of existing diversity of sampled environments (Table S1)^19^ - referred to as HQ MAGs in this study. For PD estimation alone, 2,223 medium quality (MQ) MAGs was also included.

#### Phylogenetic diversity (PD) estimation

Universally conserved single-copy marker proteins, RpoB and Ribosomal protein L1 were used for construction of a maximum likelihood phylogenetic tree and estimating total phylogenetic diversity of isolates, MAGs and metagenomes. Marker genes for RpoB were detected with multiple Pfam domains (pf04560 RNA_polymerase_Rpb2_domain_7, pf04561 RNA_polymerase_Rpb_domain_2, pf04563 RNA_polymerase_beta_subunit, pf04565 RNA_polymerase_Rpb2_domain_3 and pf00562 RNA_polymerase_Rpb2,_domain_6) assigned by the IMG annotation pipeline^14^ (that employs hmmsearch^96^), aligned with hmmalign,^97^ and individual domain alignments were concatenated into one cohesive RpoB alignment. Only sequences that covered ≥70% of the total model positions were included in tree building using Fasttree2 (LG model).^98^ Markers for ribosomal protein L1 were similarly detected (with pf00687 Ribosomal_L1), aligned and treed. For markers arising from metagenomic sequences, a minimum scaffold length of 5 kb was imposed and Actinobacterial marker sequences were identified using pplacer^99^ to place candidate sequences on a reference tree including non-actinobacterial marker genes for tree rooting and removing non-actinobacterial sequences. Using this protocol, a total of 15,114 RpoB and 16,302 ribosomal L1 genes respectively, were recovered from potentially uncultivated actinobacterial genomes from 20,100 metagenome samples from diverse environments housed within the IMG database.^14^ The PD contribution of sequences from each group (public isolates, GEBA isolates, MAGs (MQ and HQ GEMs) and metagenomes) to the overall phylogenetic diversity was inferred from the ribosomal L1 and RpoB trees separately using methods described in Wu et al.^22^ Consistent results were obtained with both markers. Original code for this analysis is publicly available: https://doi.org/10.5281/zenodo.7058177. Alignment and tree files for RpoB are available in Treebase (http://purl.org/phylo/treebase/phylows/study/TB2:S29629).

#### Biosynthetic gene cluster analysis

Secondary metabolite encoding BGC regions were identified using AntiSMASH (v6) with default settings,^44^ and ignoring contigs with lengths shorter than 5 kb. Gene cluster family (GCF) assignment for each BGC region was determined using BiG-SLICE with default settings.^100^ Potential HGT-derived BGCs were predicted by mapping genes against a list of HGT-derived genes predicted by HGTector that targets atypically distributed genes.^58^ Other horizontally acquired BGCs were identified by their location on plasmid scaffolds. Three software prediction tools were utilized to identify putative plasmid scaffolds - plasmidVerify,^101^ PlasFlow^102^ and PlasClass.^103^ These three tools employ different types of machine-learning-based classifiers (naïve Bayes, neural-network, or logistic regression, respectively) and were trained on two types of features - either plasmid-specific gene signatures (plasmidVerify) or nucleotide signatures (PlasFlow and PlasClass), thus using all three provided a robust way to identify a diverse set of plasmid scaffolds. The final set of predicted plasmid scaffolds was delineated based on overlapping predictions from at least two methods, and a minimum scaffold length of 2.5 kb (based on previous report of Actinobacterial plasmid lengths^104^).

#### Genome comparisons

For whole genome comparisons, isolate or MAG genomes were carefully selected from the IMG database using available metadata fields pertaining to isolation source or manually curated when possible. Comparisons of gene counts for individual Pfams, Tigrfams or KO terms between members of each set or group of isolates were performed. For host (2,650 genomes including 678 MAGs) versus environment (2,306 including 284 MAGs) comparisons, host genomes were smaller on average than environmental isolates (Figure S2D), therefore analyses were based on gene presence versus absence rather than gene copy number or relative abundances. Since overall taxonomic composition of each set was also highly varied or relatively biased (such as a preponderance of members of class *Acidimicrobiia* in the environmental group or class *Coriobacteriia* in the host group) (Figure S1), a phylogeny-normalized generalized linear model (GLM) approach was employed^105^ using a pairwise distance matrix based on phylogenetic distances computed from the RpoB marker tree (mentioned above), in order to minimize potentially confounding effects arising from a biased phylogenetic signal. Significant results from both a fixed and a mixed model were utilized, since occasionally, the mixed effects model fitting procedure failed to converge on a reasonable fit due to imbalances in the distribution of underlying taxa (throwing an error reported as “n/a '' in the results table). Most significant features were delineated using a false discovery rate (FDR) adjusted p-value cutoff of <0.005, and positive or negative regression coefficients (which capture magnitude of the fold differences between each group as well as the phylogenetic regression), reflect overrepresentation in host group versus environmental group, respectively (Table S3). KO and Pfam (accounting for >80% coverage (on average) of total CDS) were used to complement and validate results arising from any single function annotation type, and to examine metabolic pathways more closely (KO pathways). Custom code used for this analysis is available: https://doi.org/10.5281/zenodo.7058201.

#### Antimicrobial peptide cloning and inhibition assay

A 102 amino acid AMP candidate from *Streptosporangium becharense* DSM 46887 (GenBank ID: MBB5817359.1) with a predicted molecular weight of 11.2 kDa, pI of 8.67 and charge of 3.5, was cloned in *E. coli* for functional validation. The sequence was codon-optimized and sent to Twist Biosciences for synthesis and cloned into a pET-21(+) expression vector. The plasmid DNA (pET-21(+)_AMP_S.be) was then transferred into *E. coli* BL21(DE3) strain with 100 μg/mL carbenicillin (MilliporeSigma, Burlington, MA, USA) selection. To overexpress the short peptide, 100 mL of *E. coli* BL21(DE3) harboring the recombinant plasmid was cultured in Luria-Bertani broth containing 100 μg/mL of carbenicillin at 37°C until the mid-exponential phase. The overexpression was induced by addition of 0.1 mM isopropyl-β-D-thiogalactopyranoside (MilliporeSigma) at 25°C and 120 rpm for 16 h. The cells were harvested by centrifugation at 6,000 rpm and 4°C for 10 min and protein was extracted with BugBuster® Protein Extraction Reagent (MilliporeSigma) following the protocol from the kit. The protein was concentrated using two different Amicon Ultra centrifugal filters (MWCO 3 kDa and 30 kDa, MilliporeSigma).

Yeast *Saccharomyces cerevisiae* was used for the antimicrobial activity assessment with proteins extracted from the recombinant *E. coli. S. cerevisiae* was streaked and cultivated on the YPD agar plate at 30°C one day prior to the assay. One or a few yeast colonies were resuspended in 2 mL of 0.85% sterile saline with a sterile inoculating loop. A sterile swab was dipped into the inoculum tube and was rotated to remove the excess fluid. The swab was then streaked on the YPD plate while rotating to distribute the inoculum evenly. 6-mm sterilized filter paper disk soaked with 20–40 μL of the protein extract was placed on the YPD agar plate. Fluconazole (25 μg/disk) was used for positive control. The protein extract obtained from *E. coli* BL21(DE3) harboring empty pET-21(+) plasmid was used as negative control. After incubation at 30°C for 24 h, the antimicrobial activity of the short peptide was determined by appearance of the zone of inhibition. To confirm the overexpression of the short peptide, the protein extract was analyzed using SDS-PAGE gel (12% Mini-PROTEAN® TGX™ Precast Gel, Bio-Rad Laboratories, Hercules, CA, USA). The same amount (20 μg) of each protein extract from *E. coli* BL21(DE3) harboring the recombinant plasmid or empty plasmid was loaded in a single well and the gel was run at 180 V for 40 min. The gel was stained using Coomassie Brilliant Blue R-250 Staining Solution (Bio-Rad Laboratories).

#### Prophage detection

The 5,648 isolate genomes were screened for potential prophages using VirSorter 2.1,^65^ using the dsDNAphage, RNA, and ssDNA models, a minimum score of 0.7, and a minimum length of 1 kb, as well as the Inovirus Detector scripts (v2019-06-30) with default parameters.^106^ Predicted prophages were then analyzed for completeness and contamination with CheckV 0.7.0^66^ using the end_to_end option and default parameters otherwise. Prophage prediction corresponding to common contamination/errors were identified based on the CheckV results and the VirSorter2 functional annotations as follows: all genes from the predicted prophages are similar to gene from Type 6 Secretion Systems; the predicted prophages is only composed of ≥3 contiguous peptidase genes; CheckV detects ≥1 host marker and 0 viral marker in the prophage; CheckV detects ≥2 host markers and ≤1 viral marker in the prophage; CheckV detects a host region of ≥2 genes. For all potential contamination detected via CheckV, the predicted prophage region was trimmed to the CheckV-predicted viral region and/or the nearest integrase(-like) gene within 5 kb of the predicted ends of a CheckV-predicted viral region. The gene content and VirSorter2 annotation of all prophages predicted to be ≥150% complete and/or with a length ≥50 kb were inspected, and the prophage boundaries were adjusted when the initial prediction included two contiguous but distinct prophages. Finally, to avoid including partial prophages, only predicted prophages predicted to be ≥50% complete or ≥10 kb (or ≥ 1 kb for inoviruses) were retained.

Selected prophages were next clustered at 95% average nucleotide identity (ANI) and 85% alignment fraction (AF) using ClusterGenomes v5.1 (https://github.com/simroux/ClusterGenomes) and only the largest representative of each cluster was retained.^106^ From this non-redundant set, predicted prophages with a CheckV completeness estimation of ≥75% were considered as “(near-)complete”, along with predicted prophages with surrounding host regions of ≥ 5 kb on both 5′ and 3′ ends except if these had a high-confidence CheckV completeness estimation <75% or if CheckV provided no completeness estimation at all, the latter typically corresponding to partial prophages too short for CheckV to estimate completeness. This was based on the observation that predicted prophages with a high-confidence CheckV completeness estimation and with surrounding host regions of ≥ 5 kb on both 5′ and 3′ ends were overwhelmingly (89.6%) estimated to be ≥75% complete. To evaluate the distribution and diversity of *Actinobacteria* prophages, predicted proteins from *Actinobacteria* genomes were compared to the predicted proteins from the non-redundant prophage set using Diamond v0.9.25 in “--sensitive” mode.^107^ A prophage protein was considered as detected in an *Actinobacteria* genome if covered by a hit with ≥90% identity and ≥90% coverage. A prophage was considered as detected in an *Actinobacteria* genome if ≥ 50% of its predicted proteins were detected, and a (near-)complete prophage was considered as entirely detected in a genome if ≥ 90% of its predicted proteins were detected. Potential link between the detection of prophages and other genome features, including taxonomy, environment, presence of a BGC, and number of scaffolds in the genome (≤5 or >5) was evaluated via ANOVA performed in R v4.1 (function aov^108^). All genomes for which these features were unknown were excluded from the analysis. Each taxonomic rank (genus, family, order, and class) was evaluated separately, and each time only taxa with ≥20 genomes were included in the analysis.

The diversity of prophages identified in *Actinobacteria* genomes was evaluated through a vContact2 v 0.9.11 genome network clustering.^73^ For this analysis, 14,256 reference phage genomes from NCBI RefSeq and GenBank collected using the INfrastructure for a PHAge Reference Database perl script (https://github.com/RyanCook94/inphared.pl; data downloaded on 01/21/2021) were clustered along with the 3,393 representative *Actinobacteria* prophages considered as “near-complete” (see above). The viral clusters (“VCs”) identified by vContact2 and including at least one *Actinobacteria* prophage were then interpreted as “genus-level” groups when evaluating the diversity of prophages associated with different *Actinobacteria* host taxa.

Finally, the gene content of insertion sites/regions was evaluated in *Actinobacteria* prophages based on IMG genome annotation. The predicted functions of prophage-encoded genes situated within 1 kb of the prophage predicted boundaries, i.e., the last few genes encoded by the prophage before its 5′ and 3′ ends, were searched for canonical insertion sites and prophage edge genes including tRNA and integrase-like genes (annotated as “integrase”, “recombinase”, or “excisionase”). Prophages were classified as “tRNA and integrase-like”, “tRNA”, “integrase-like”, or “other” ends based on the presence of a tRNA and an integrase-like gene, a tRNA gene only, an integrase-like gene only, or neither a tRNA nor an integrase-like gene within the 1 kb edge of a prophage. Similarly, regions within 1 kb of the prophage predicted boundaries, i.e., the 1 kb regions immediately outside of the prophage 5′ and 3′ ends, were searched for tRNA, DNA binding/transcriptional regulator genes (i.e., genes annotated “transcription regulator”, “transcription activator”, “gntR”, “acrR”, “tetR”, “HTH”, and “DNA-binding”), transposase genes, and integrase-like genes.

## Data Availability

• All genome data generated in this study are publicly available in GenBank and IMG (individual accession numbers are listed in Table S1). • All original code has been deposited at Zenodo and is publicly available. DOIs are listed in the key resources table. • Alignment and tree files used for PD estimation have been deposited in Treebase: http://purl.org/phylo/treebase/phylows/study/TB2:S29629.

## Supplemental information

Document S1. Figures S1–S17 and Data S1 and S2

Table S1. List of GEBA-Actino genomes, genome statistics and metadata, environmental metadata, and more; includes a second worksheet with the list of 4,824 Public isolate genomes and 1,098 MAGs, related to Figures 1, 2, S1, and S2

Table S2. List of metagenome samples contributing RpoB markers for clades poorly or unrepresented by isolates, related to Figure 1C

Table S3. Significant results from comparing host-associated versus environmental genomes using phylogeny-normalized generalized linear model, related to Figures 2A and 2B Results based on counts of genes assigned to Pfams and KEGG Orthology (KO) terms are shown in individual worksheets.

Table S4. Significant results from comparing plant-host versus animal-host isolate genomes from the order Micrococcales, related to Figures 2A and 2B Functional differences are based on Tigrfam assignments of CDS of members in each group.

Table S5. Complete list of biosynthetic gene clusters (BGCs) predicted by AntiSMASH from all isolate genomes in this study, related to Figures 3, 4, and S9 Summarized results per genome and a separate list of genomes that had no predicted BGCs are provided in additional worksheets.

Table S6. List of gene cluster families (GCFs) computed from BGCs using BiG-SLiCE, related to Figures 3A, S6A, and S6B

Table S7. List of predicted plasmid scaffolds from all isolate genomes in this study, related to Figures 4A, S12, and S13

Table S8. List of prophage-like regions in isolate genomes, related to Figures 5, S15, and S16

Document S2. Article plus supplemental information
