# Supplementary material for: Expanding the genomic encyclopedia of Actinobacteria with 824 isolate reference genomes
Source: Cell Genom. 2022 Nov 11;2(12):100213. doi: 10.1016/j.xgen.2022.100213 (PMC9903846; doi:10.1016/j.xgen.2022.100213)
Supplement: Document S1. Figures S1–S17 and Data S1 and S2 [file mmc1.pdf]

Cell Genomics, Volume *2*

Supplemental information

Expanding the genomic encyclopedia of *Actinobacteria* with 824 isolate reference genomes

Rekha Seshadri, Simon Roux, Katharina J. Huber, Dongying Wu, Sora Yu, Dan Udwary, Lee Call, Stephen Nayfach, Richard L. Hahnke, Rüdiger Pukall, James R. White, Neha J. Varghese, Cody Webb, Krishnaveni Palaniappan, Lorenz C. Reimer, Joaquim Sardà, Jonathon Bertsch, Supratim Mukherjee, T.B.K. Reddy, Patrick P. Hajek, Marcel Huntemann, I-Min A. Chen, Alex Spunde, Alicia Clum, Nicole Shapiro, Zong-Yen Wu, Zhiying Zhao, Yuguang Zhou, Lyudmila Evtushenko, Sofie Thijs, Vincent Stevens, Emiley A. Eloe-Fadrosh, Nigel J. Mouncey, Yasuo Yoshikuni, William B. Whitman, Hans-Peter Klenk, Tanja Woyke, Markus Göker, Nikos C. Kyrpides, and Natalia N. Ivanova

SUPPLEMENTARY FIGURES

Figure S1. Genome counts by taxon (class) for isolate groups (GEBA-Actino and Public) or MAGs (see methods) analyzed in this study. Related to Figure 1.

CLASS	GEBA	PUBLIC	MAG	Total
Actinobacteria	813	4585	622	6020
Coriobacteriia	5	210	265	480
Acidimicrobiia	3	8	163	174
Thermoleophilia	3	9	34	46
Rubrobacteria		6	1	7
Nitriliruptoria		6		6
Unclassified			13	13
**Total**	**824**	**4824**	**1098**	**6746**

Figure S2. Genome counts and size statistics for groups analyzed in this study. a, Distribution of genome sizes for 824 GEBA-Actino isolates is contrasted with 4824 public isolates and 1098 MAGs. See Table S1 for complete genome lists and associated environmental and sequencing metadata. **b,** Genome counts by isolation source ecosystem for isolate (GEBA-Actino or Public) groups or metagenome sample habitat for MAGs analyzed in this study. **c,** Genome size distribution by taxon (class) of all genomes analyzed in this study. **d,** Genome size distribution by ecosystem. **e**, Genome size distribution by dataset type and taxon (class). **f,** Genome size distribution by dataset "type" and ecosystem. Related to Figures 1- 3.

A

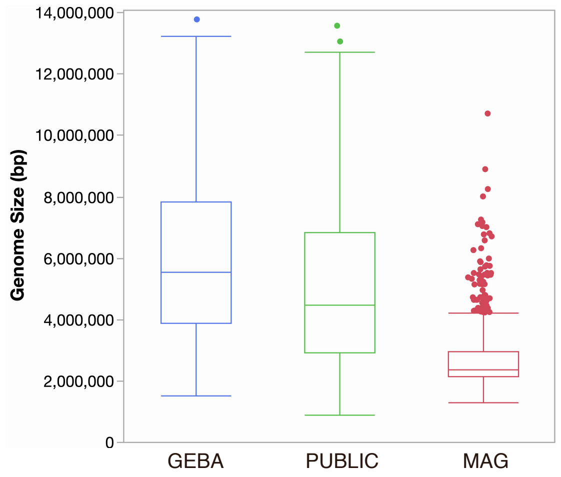

B

Ecosystem	GEBA	PUBLIC	MAG	Total
Host-associated	240	2693	705	3638
Terrestrial	402	1050	93	1545
Aquatic	91	474	191	756
Engineered	64	305	108	477
Unknown	24	296		320
Air	3	6	1	10
**Total**	**824**	**4824**	**1098**	**6746**

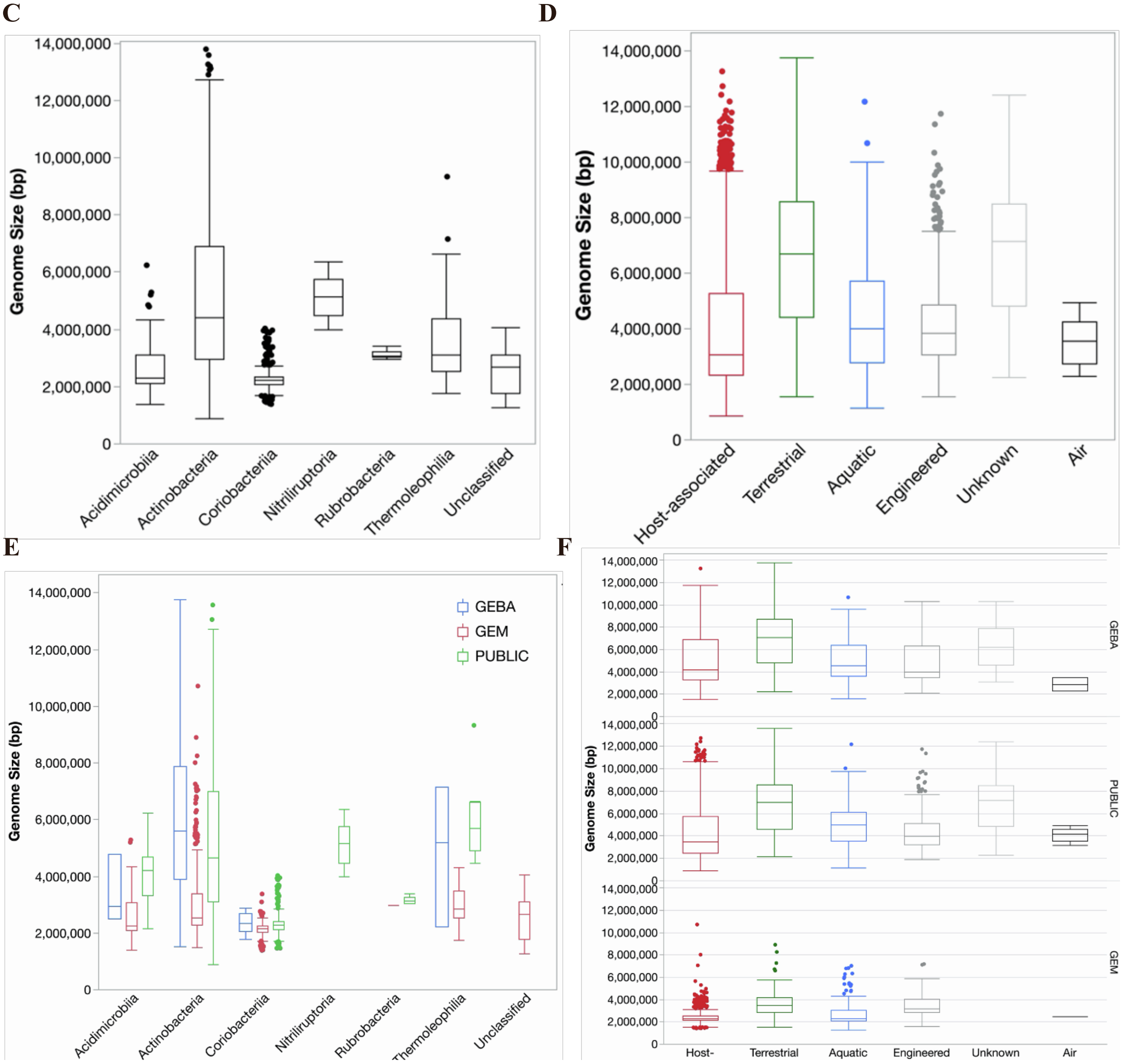

Figure S3. Total estimated phylogenetic diversity of (a) Class *Actinobacteria* and (b) Class *Coriobacteriia* (the two most populous classes based on available isolate genome numbers). Public in green and GEBA-Actino in blue, MAGs (HQ in red and MQ in orange), and metagenomic sequences uncaptured in MAGs, in gray. Related to Figure 1bc.

A

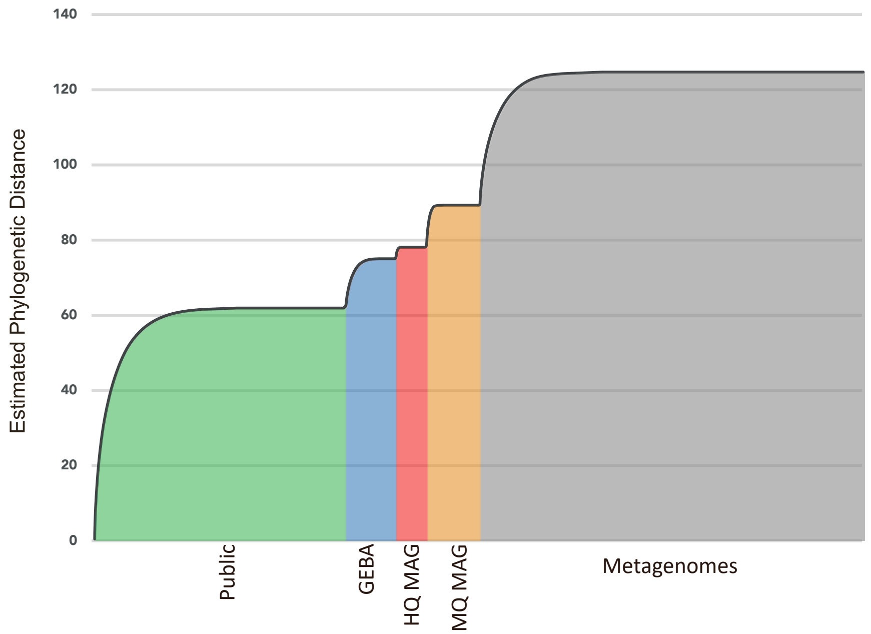

B

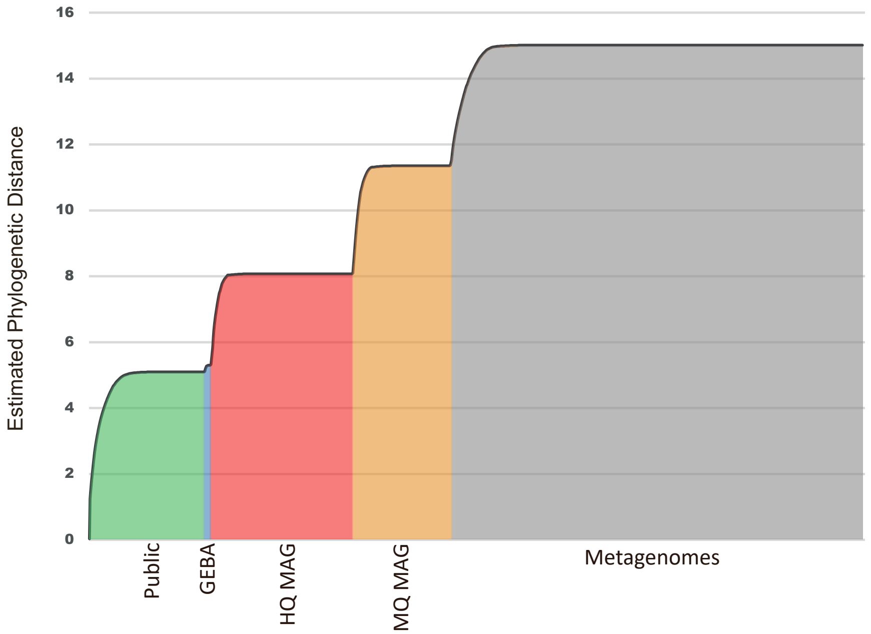

Figure S4. Examples of Pfams with candidate genes displaying a limited phylogenetic distribution. Related to Figure 2ab.

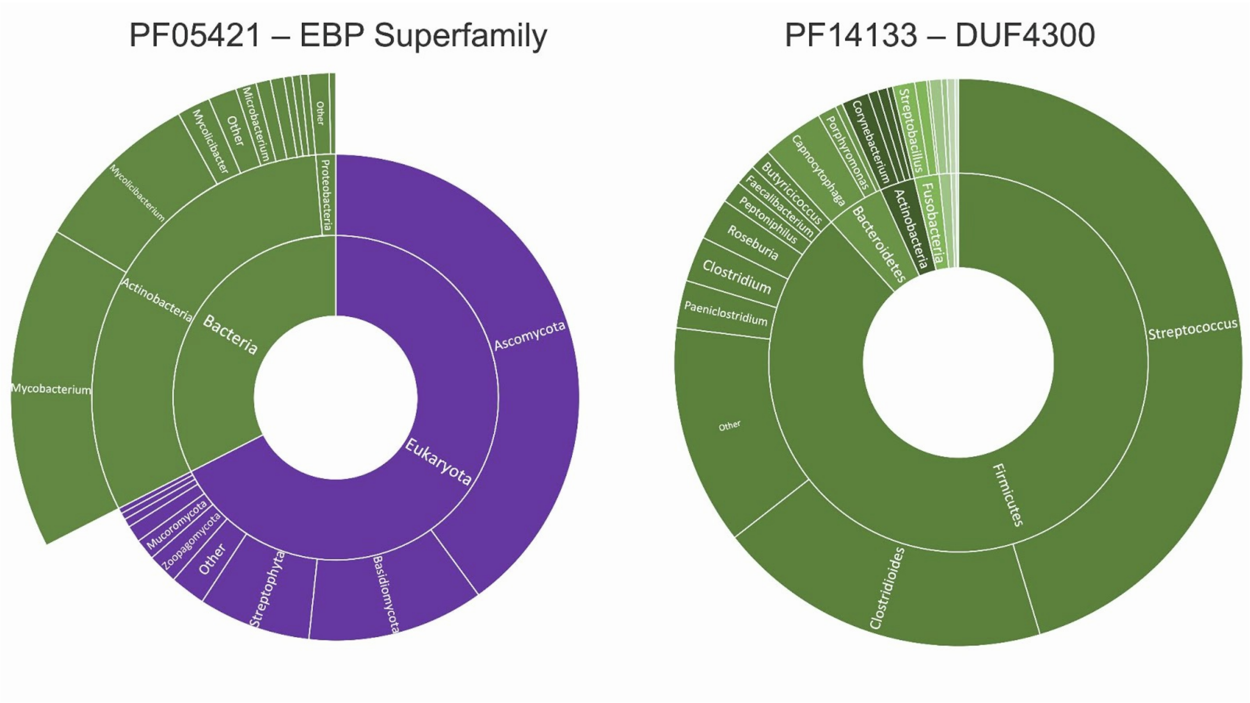

Figure S5. Maximum likelihood tree of a subset of bacterial candidate sequences assigned to PF04916 (putative phospholipase B). Tree branches are colored by Phylum. The taxonomically incongruent placement of taxa in this gene tree is exemplified by the presence of the *Lactobacillus* spp. (Phylum *Firmicutes*) sub-clade within the *Bifidobacterium* spp. (Phylum *Actinobacteria*) clade. Members of both these genera are almost exclusively found in the mammalian digestive system. Related to Figure 2ab.

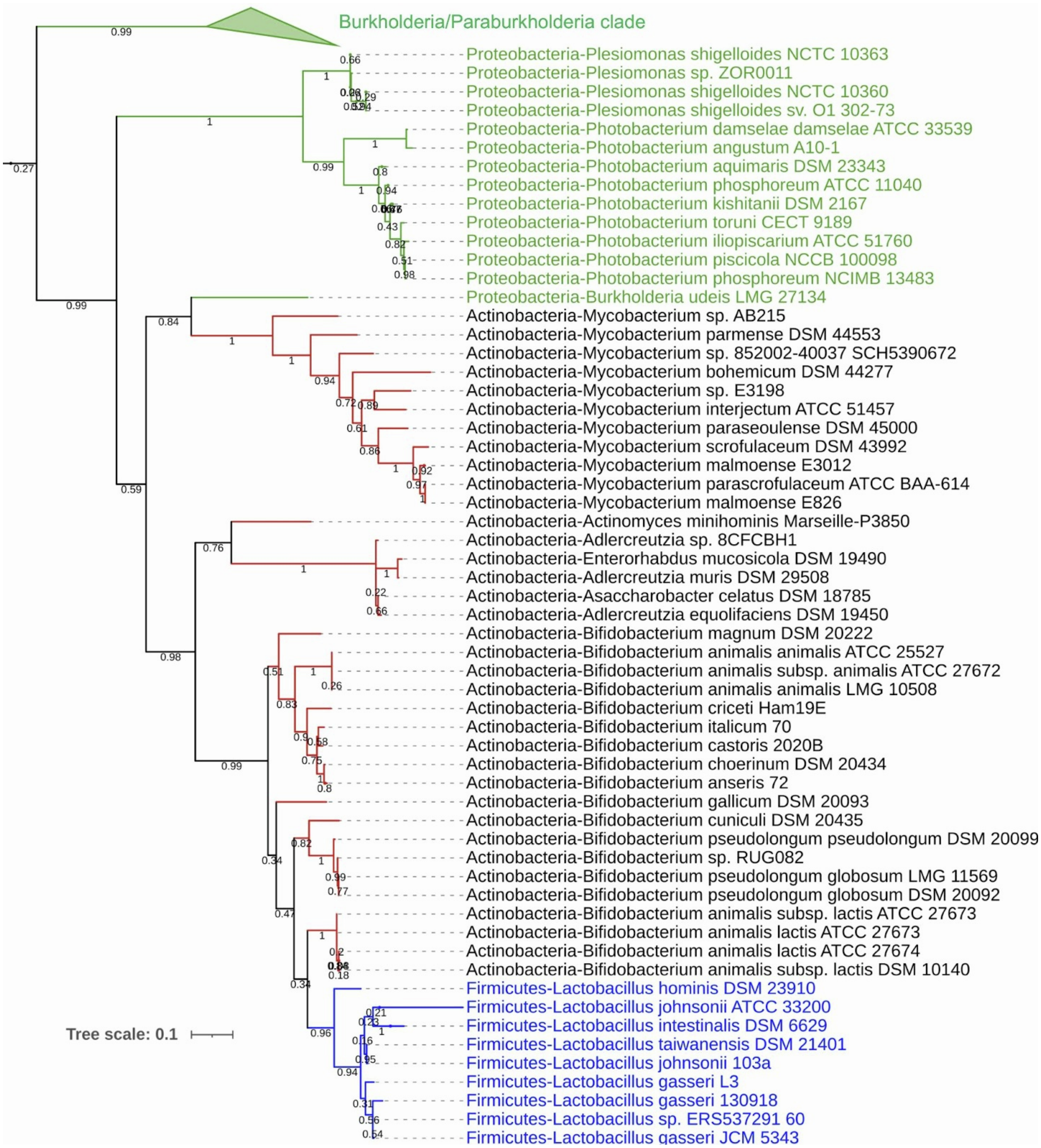

Figure S6. a, Size distribution of total GCFs. **b**, Largest GCFs are shown, X-axis indicates the SM class of BGCs in each GCF and Y-axis indicated total number of BGCs in each cluster. Related to Figure 3a.

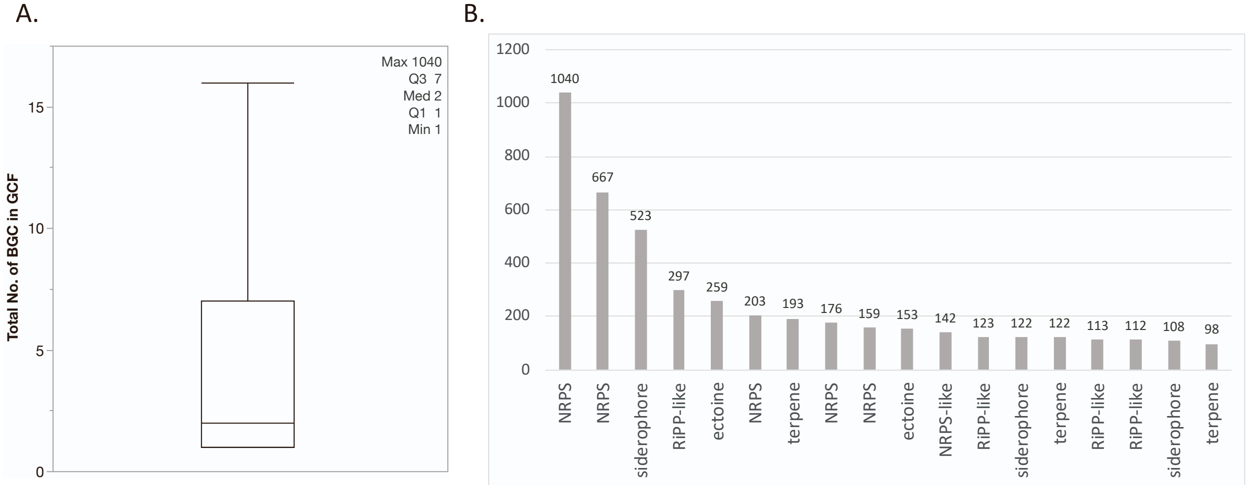

Figure S7. Comparing the distribution of %BGC of isolate genomes by broad ecosystem categories. Isolates including GEBA-Actino and Public genomes and categorization is based on isolation source - host-associated (includes no plant-associated genomes) versus environmental (e.g., terrestrial, aquatic, and excluding "engineered" genomes). Five quantile text summaries are included for each boxplot. Related to Figure 3.

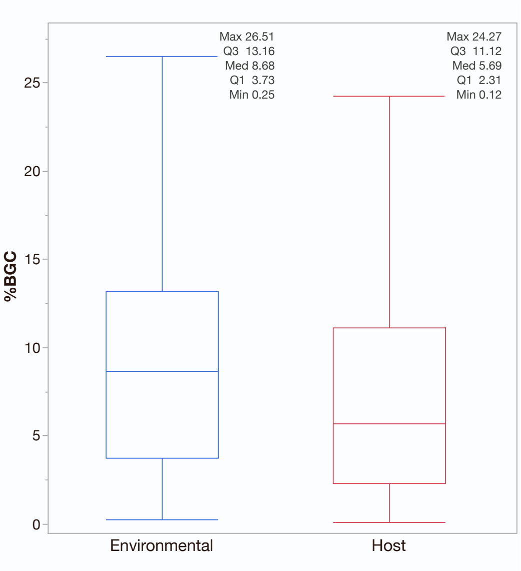

Figure S8. Distribution of %BGC (Y-axis) of isolates by taxonomic level (a) Class (total number of genomes in each class shown in parentheses) **(b)** Order, colored by Class, ordered by descending mean %BGC **(c)** Genus (only top 95 shown). Related to Figure 3.

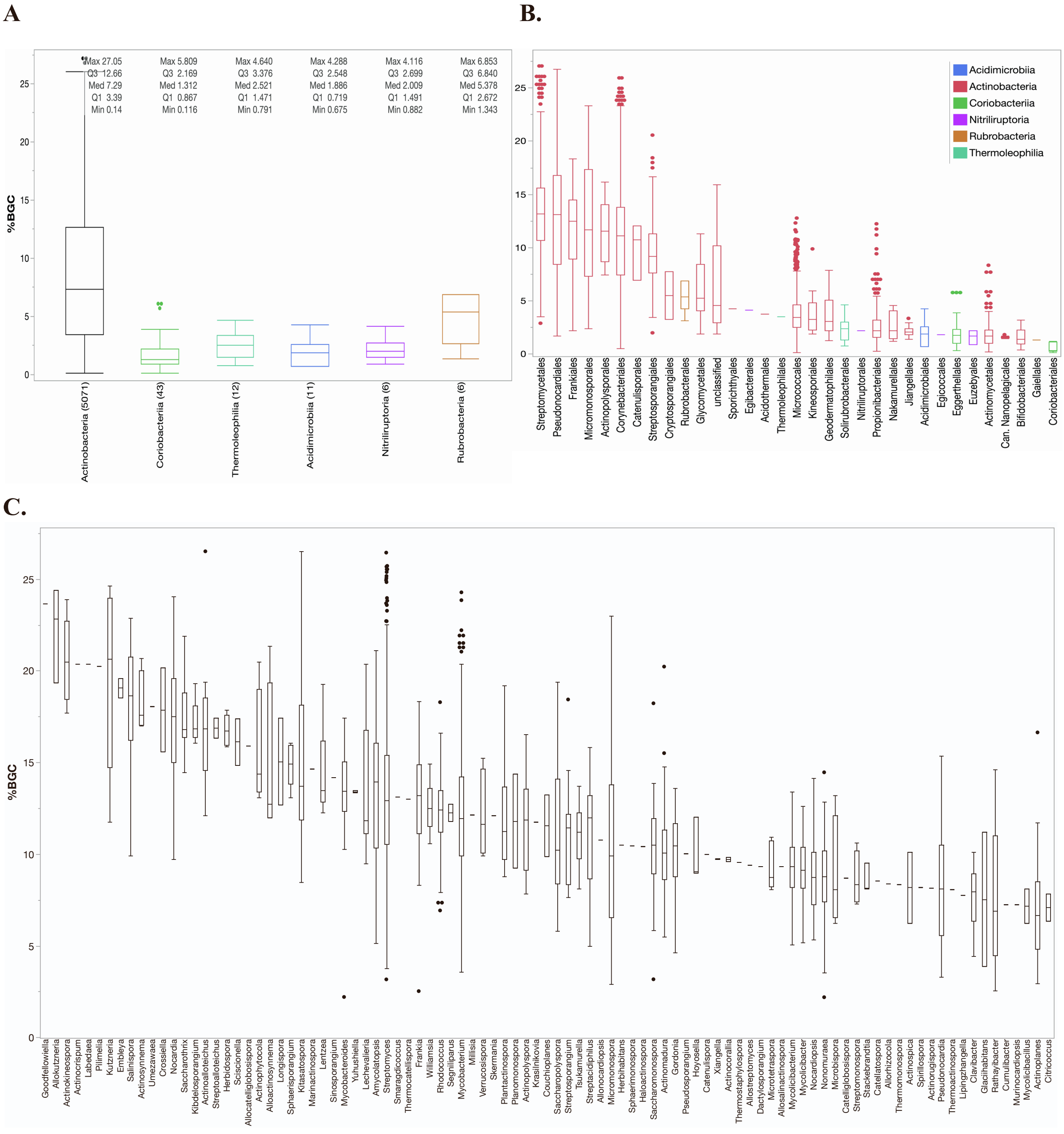

Figure S9. Genome sizes of isolates (GEBA-Actino or Public) with no predicted BGCs. Genus names are shown on the X-axis and genome size on Y-axis. Color denotes isolation source where red is host-associated, green is terrestrial, and grey is unknown or engineered. Related to Figure 3.

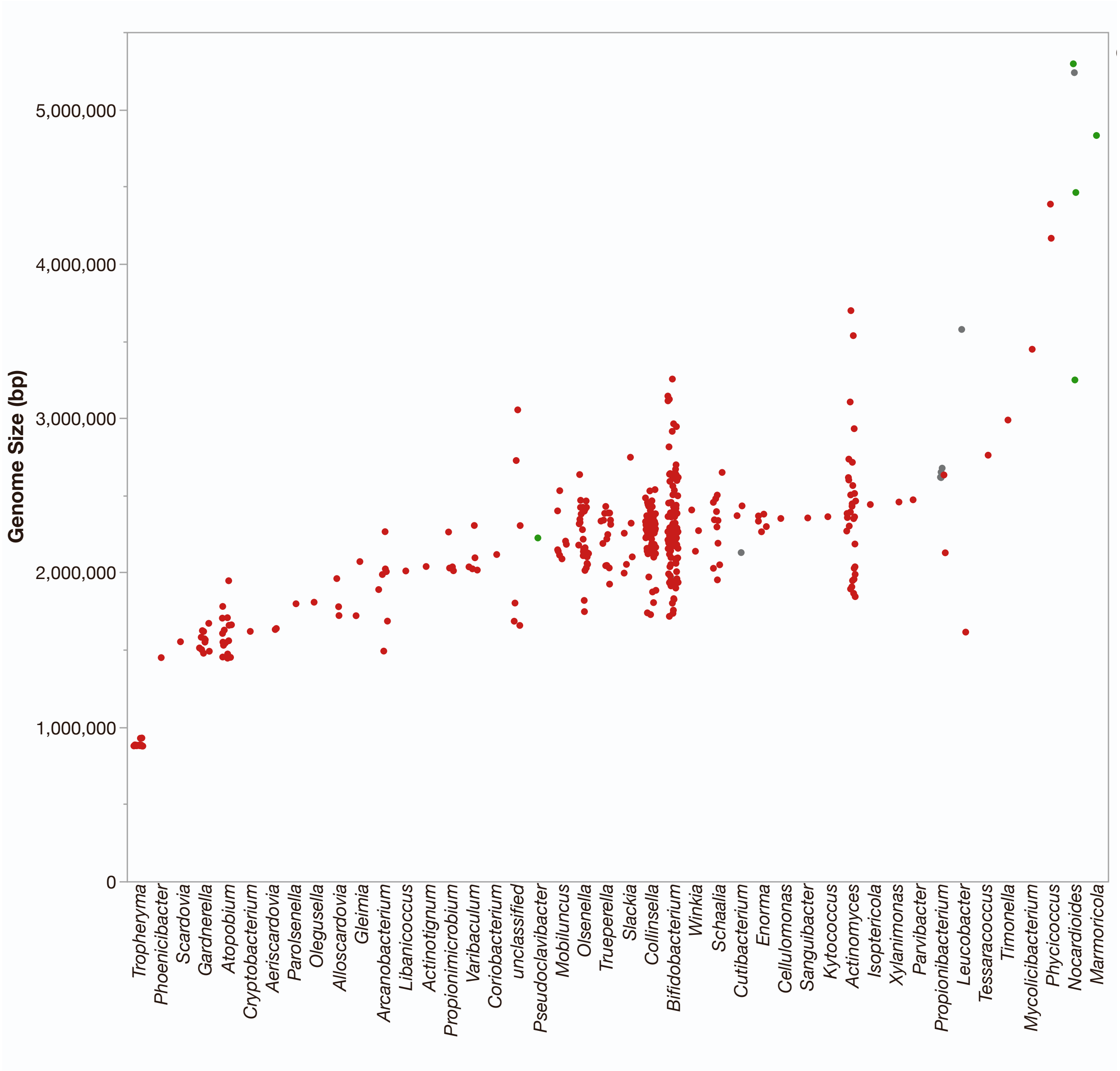

Figure S10. Maximum likelihood RpoB tree of strains of *Gardnerella vaginalis* annotated with presence (red) or absence (uncolored) of the solitary BGC (a type III PKS) predicted in this lineage. Related to Figure 4.

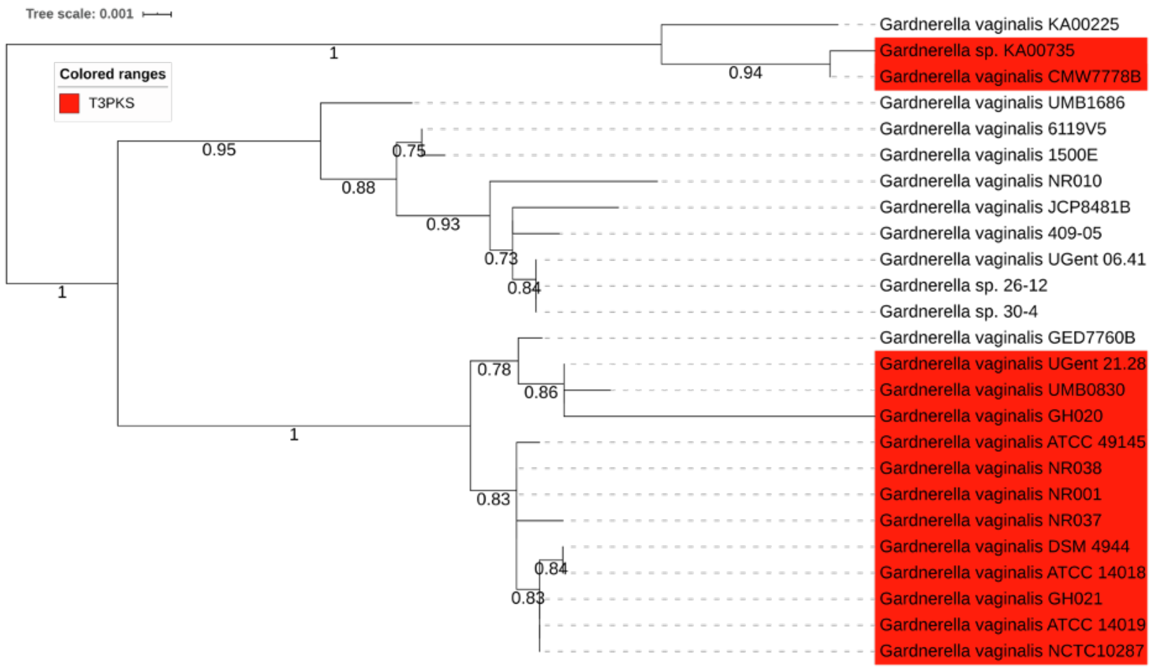

Figure S11. Disjointed BGC distribution in *Bifidobacterium* spp. a, Maximum likelihood RpoB tree of *Bifidobacterium* spp. annotated with predicted BGCs. Sub-clades are highlighted with boxes as discussed in the main text. Members of collapsed clades typically had no BGC except for one strain of *B. animalis* (MCC 0499) with a single NRPS-like pediction. Inactivation of a lanthipeptide hook protein in individual strains is depicted with a "☠". BGCs appear in columns numbered 1 to 20: 1. RiPP-like, 2. phenazine, 3. RRE-containing, 4. lanthipeptide-class-ii, 5. lanthipeptide-class-iv, 6. T3PKS, 7. hglE-KS, 8. lanthipeptide-class-iii_RRE-containing, 9. lanthipeptide-class-iii, 10. NRPS, 11. terpene, 12. lanthipeptide-class-iii_lassopeptide, 13. LAP, 14. lanthipeptide-class-i, 15. NRPS_RRE-containing, 16. NRPS-like, 17. thiopeptide, 18. lanthipeptide-class-iv_RRE-containing, 19. LAP_thiopeptide, and 20. Lassopeptide. BGCs with a ★ may have been acquired by horizontal gene transfer (as discussed in the main text). **b (inset)**, Inactivation of the lanthipeptide hook protein (yellow arrows) in specific *Bifidobacterium pseudocatenulatum* strains (DC2A and DSM 20438) is contrasted to intact orthologs in other closely related strains. Green arrows depict other constituents of the BGC region identified by AntiSMASH. Related to Figure 4.

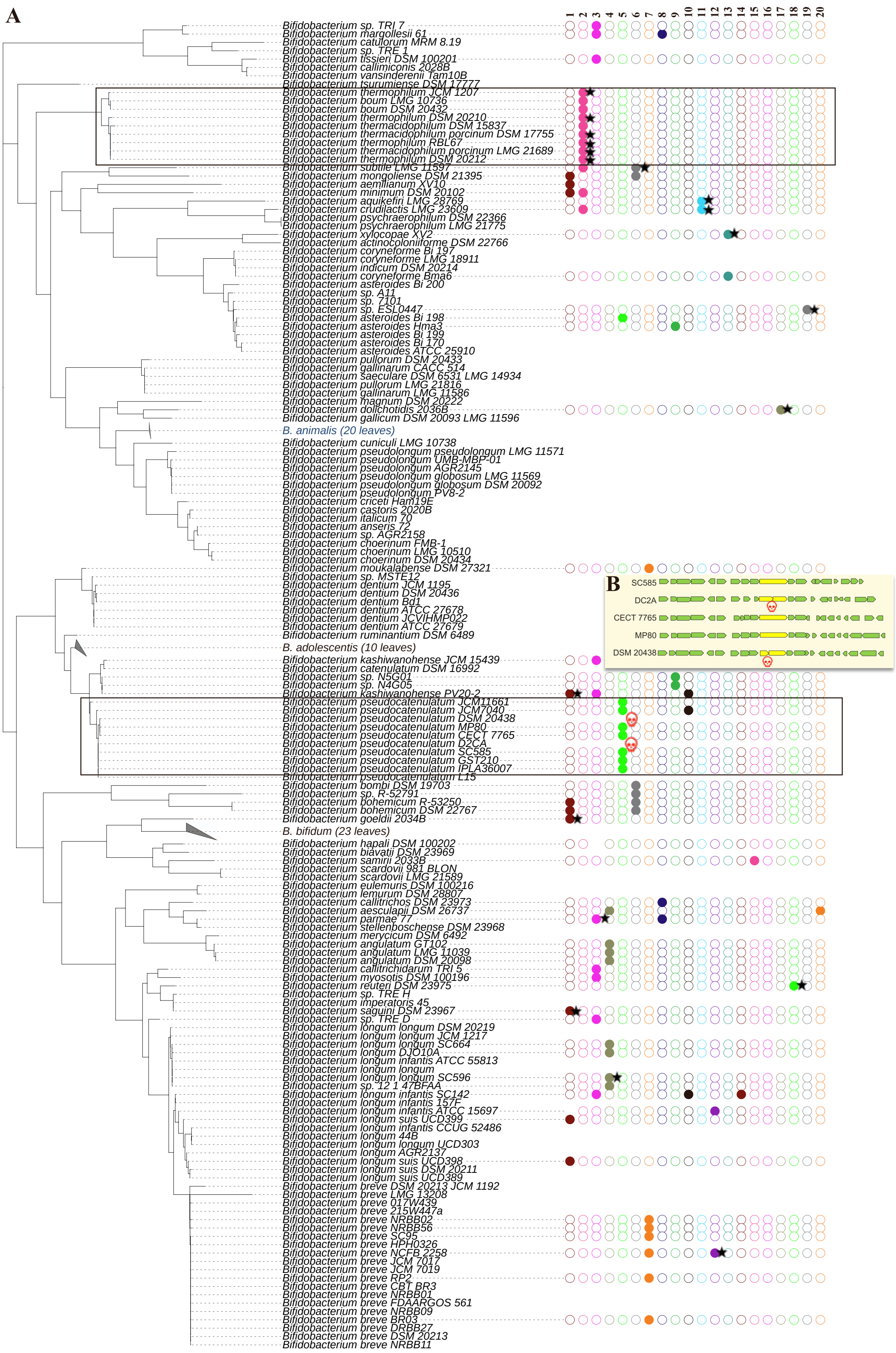
A
1 2 3 4 5 6 7 8 9 10 11 12 13 14 15 16 17 18 19 20
Bifidobacterium sp. TRI 7
Bifidobacterium margollesii 61
Bifidobacterium catulorum MRM 8.19
Bifidobacterium sp. TRE 1
Bifidobacterium tissieri DSM 100201
Bifidobacterium callimiconis 2028B
Bifidobacterium vansinderenii Tam10B
Bifidobacterium tsurumiense DSM 17777
Bifidobacterium thermophilum JCM 1207
Bifidobacterium boum LMG 10736
Bifidobacterium boum DSM 20432
Bifidobacterium thermophilum DSM 20210
Bifidobacterium thermacidophilum DSM 15837
Bifidobacterium thermacidophilum porcinum DSM 17755
Bifidobacterium thermophilum RBL67
Bifidobacterium thermacidophilum porcinum LMG 21689
Bifidobacterium thermophilum DSM 20212
Bifidobacterium subtile LMG 11597
Bifidobacterium mongoliense DSM 21395
Bifidobacterium aemilianum XV10
Bifidobacterium minimum DSM 20102
Bifidobacterium aquikefiri LMG 28769
Bifidobacterium crudilactis LMG 23609
Bifidobacterium psychraerophilum DSM 22366
Bifidobacterium psychraerophilum LMG 21775
Bifidobacterium xylocopae XV2
Bifidobacterium actinocoloniiforme DSM 22766
Bifidobacterium coryneforme Bi 197
Bifidobacterium coryneforme LMG 18911
Bifidobacterium indicum DSM 20214
Bifidobacterium coryneforme Bma6
Bifidobacterium asteroides Bi 200
Bifidobacterium sp. A11
Bifidobacterium sp. 7101
Bifidobacterium sp. ESL0447
Bifidobacterium asteroides Bi 198
Bifidobacterium asteroides Hma3
Bifidobacterium asteroides Bi 199
Bifidobacterium asteroides Bi 170
Bifidobacterium asteroides ATCC 25910
Bifidobacterium pullorum DSM 20433
Bifidobacterium gallinarum CACC 514
Bifidobacterium saeculare DSM 6531 LMG 14934
Bifidobacterium pullorum LMG 21816
Bifidobacterium gallinarum LMG 11586
Bifidobacterium magnum DSM 20222
Bifidobacterium dolichotidis 2036B
Bifidobacterium gallicum DSM 20093 LMG 11596
B. animalis (20 leaves)
Bifidobacterium cuniculi LMG 10738
Bifidobacterium pseudolongum pseudolongum LMG 11571
Bifidobacterium pseudolongum UMB-MBP-01
Bifidobacterium pseudolongum AGR2145
Bifidobacterium pseudolongum globosum LMG 11569
Bifidobacterium pseudolongum globosum DSM 20092
Bifidobacterium pseudolongum PV8-2
Bifidobacterium criceti Ham19E
Bifidobacterium castoris 2020B
Bifidobacterium italicum 70
Bifidobacterium anseris 72
Bifidobacterium sp. AGR2158
Bifidobacterium choerinum FMB-1
Bifidobacterium choerinum LMG 10510
Bifidobacterium choerinum DSM 20434
Bifidobacterium moukalabense DSM 27321
Bifidobacterium sp. MSTE12
Bifidobacterium dentium JCM 1195
Bifidobacterium dentium DSM 20436
Bifidobacterium dentium Bd1
Bifidobacterium dentium ATCC 27678
Bifidobacterium dentium JCVIHMP022
Bifidobacterium dentium ATCC 27679
Bifidobacterium ruminantium DSM 6489
B. adolescentis (10 leaves)
Bifidobacterium kashiwanohense JCM 15439
Bifidobacterium catenulatum DSM 16992
Bifidobacterium sp. N5G01
Bifidobacterium sp. N4G05
Bifidobacterium kashiwanohense PV20-2
Bifidobacterium pseudocatenulatum JCM11661
Bifidobacterium pseudocatenulatum JCM7040
Bifidobacterium pseudocatenulatum DSM 20438
Bifidobacterium pseudocatenulatum MP80
Bifidobacterium pseudocatenulatum CECT 7765
Bifidobacterium pseudocatenulatum D2CA
Bifidobacterium pseudocatenulatum SC585
Bifidobacterium pseudocatenulatum GST210
Bifidobacterium pseudocatenulatum IPLA36007
Bifidobacterium pseudocatenulatum L15
Bifidobacterium bombi DSM 19703
Bifidobacterium sp. R-52791
Bifidobacterium bohemicum R-53250
Bifidobacterium bohemicum DSM 22767
Bifidobacterium goeldii 2034B
B. bifidum (23 leaves)
Bifidobacterium hapali DSM 100202
Bifidobacterium biavatii DSM 23969
Bifidobacterium samirii 2033B
Bifidobacterium scardovii 981 BLON
Bifidobacterium scardovii LMG 21589
Bifidobacterium eulemuris DSM 100216
Bifidobacterium lemurum DSM 28807
Bifidobacterium callitrichos DSM 23973
Bifidobacterium aesculapii DSM 26737
Bifidobacterium parmae 77
Bifidobacterium stellenboschense DSM 23968
Bifidobacterium merycicum DSM 6492
Bifidobacterium angulatum GT102
Bifidobacterium angulatum LMG 11039
Bifidobacterium angulatum DSM 20098
Bifidobacterium callitrichidarum TRI 5
Bifidobacterium myosotis DSM 100196
Bifidobacterium reuteri DSM 23975
Bifidobacterium sp. TRE H
Bifidobacterium imperatoris 45
Bifidobacterium saguini DSM 23967
Bifidobacterium sp. TRE D
Bifidobacterium longum longum DSM 20219
Bifidobacterium longum longum JCM 1217
Bifidobacterium longum longum SC664
Bifidobacterium longum DJO10A
Bifidobacterium longum infantis ATCC 55813
Bifidobacterium longum longum
Bifidobacterium longum longum SC596
Bifidobacterium sp. 12 1 47BFAA
Bifidobacterium longum infantis SC142
Bifidobacterium longum infantis 157F
Bifidobacterium longum infantis ATCC 15697
Bifidobacterium longum suis UCD399
Bifidobacterium longum infantis CCUG 52486
Bifidobacterium longum 44B
Bifidobacterium longum longum UCD303
Bifidobacterium longum AGR2137
Bifidobacterium longum suis UCD398
Bifidobacterium longum suis DSM 20211
Bifidobacterium longum suis UCD389
Bifidobacterium breve DSM 20213 JCM 1192
Bifidobacterium breve LMG 13208
Bifidobacterium breve 017W439
Bifidobacterium breve 215W447a
Bifidobacterium breve NRBB02
Bifidobacterium breve NRBB56
Bifidobacterium breve SC95
Bifidobacterium breve HPH0326
Bifidobacterium breve NCFB 2258
Bifidobacterium breve JCM 7017
Bifidobacterium breve JCM 7019
Bifidobacterium breve RP2
Bifidobacterium breve CBT BR3
Bifidobacterium breve NRBB01
Bifidobacterium breve FDAARGOS 561
Bifidobacterium breve NRBB09
Bifidobacterium breve BR03
Bifidobacterium breve DRBB27
Bifidobacterium breve DSM 20213
Bifidobacterium breve NRBB11
B
SC585
DC2A
CECT 7765
MP80
DSM 20438

Figure S12. Plasmid lengths (Y-axis) distribution by taxonomic Class (X-axis). Individual data points correspond to a single scaffold and are colored based on the presence of one or more BGC (green) or absence thereof (grey). Five quantile summary of plasmid length for each class are shown on top. Related to Figure 4a.

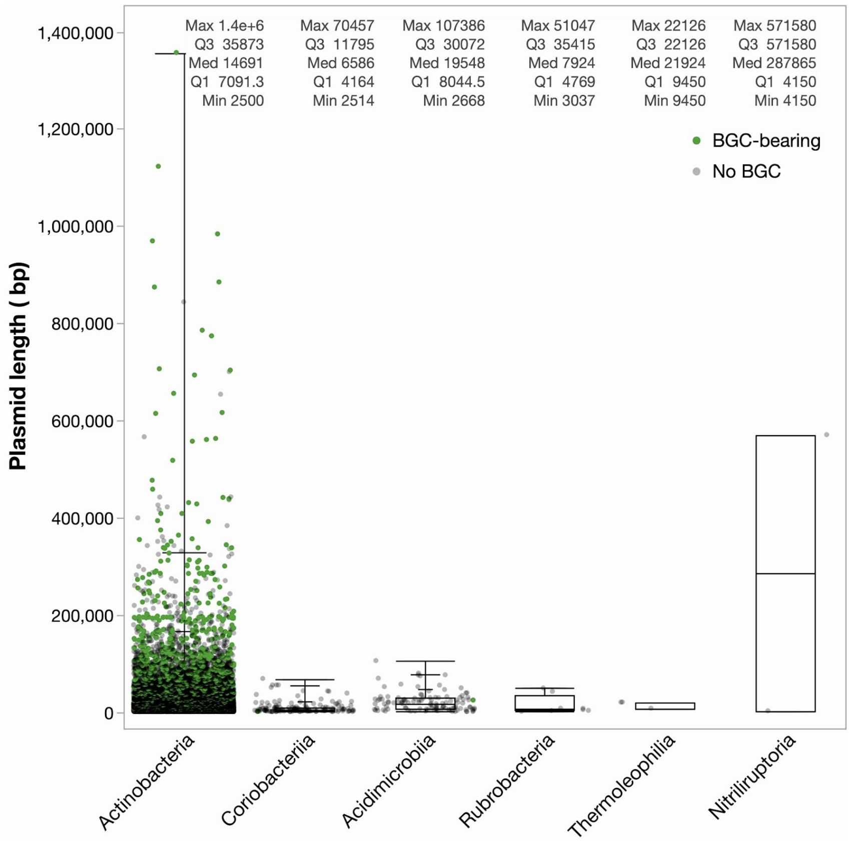

Figure S13. Taxa containing plasmid borne BGC. Genera with the highest numbers of genomes with BGC-bearing plasmids (red bar) are shown. X-axis in the individual genus name, primary Y-axis is the total number of genomes per genus and secondary Y-axis is the average % BGC for BGC-encoding genomes. Related to Figure 4a.

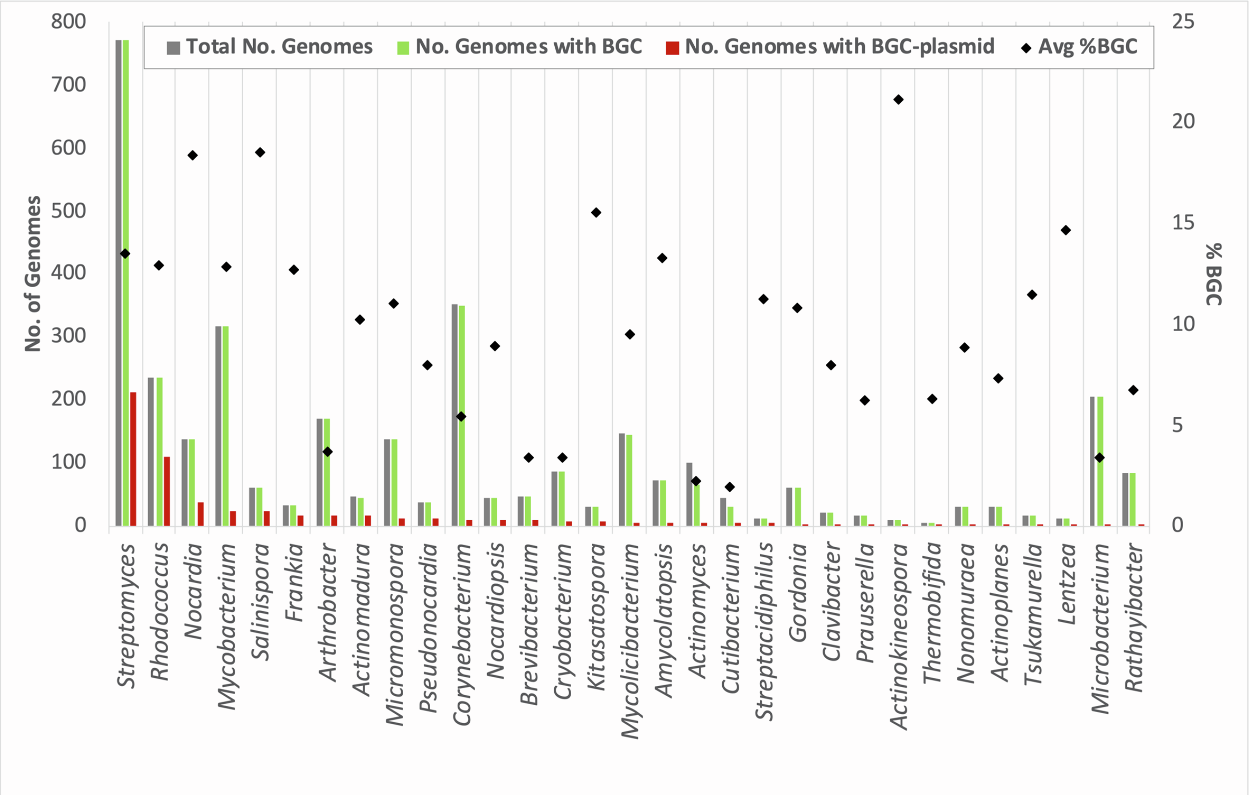

Figure S14. SM types commonly displaying potential HGT. Bar graphs contrast counts of BGCs with (orange bars)- and without (blue bars) evidence of HGT. Only BGCs with >60 total count is displayed). On the X-axis, SM class is ordered by descending order of total number of BGC per class. Related to Figure 4c.

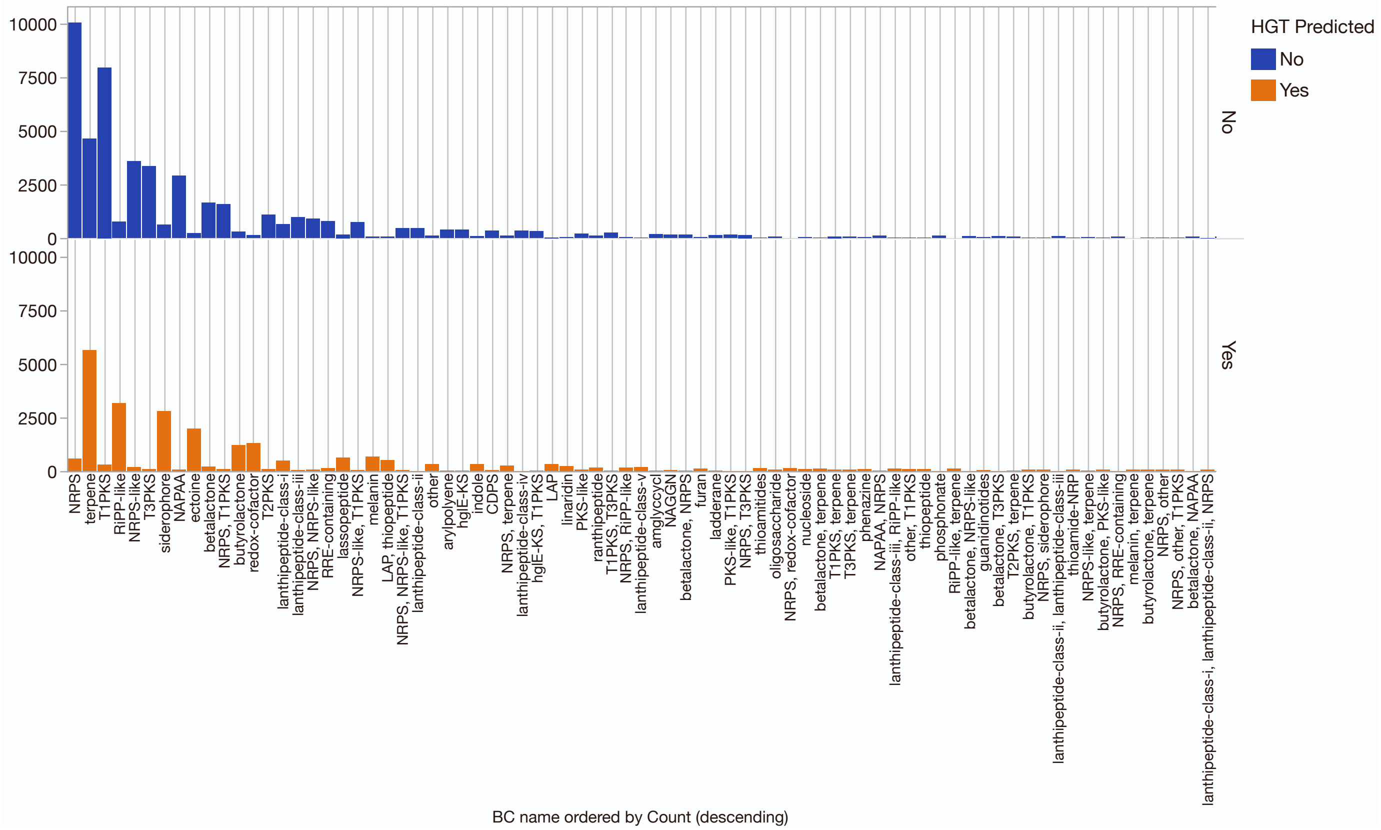

Figure S15. **Length, flanking regions, and estimated completeness of *Actinobacteria* prophages. a,** Distribution of prophage length by type, i.e. prophages with flanking host regions in 5' and 3', prophages with flanking region on only one side, and prophages without a flanking region. **b,** Estimated completeness by prophage type, only for prophages for which a high-confidence completeness estimation could be obtained with CheckV. These represented 17%, 15%, and 25% of the prophages in the "Integrated with surrounding host regions", "Integrated on contig edge", and "Contig without host region" types, respectively. Hence, for the remaining prophages without a high-confidence completeness estimation, all "Integrated with surrounding host regions" were considered as near-complete. **c,** Estimated completeness by prophage length, only for prophages for which a high-confidence completeness estimation could be obtained with CheckV. Related to Figure 5.

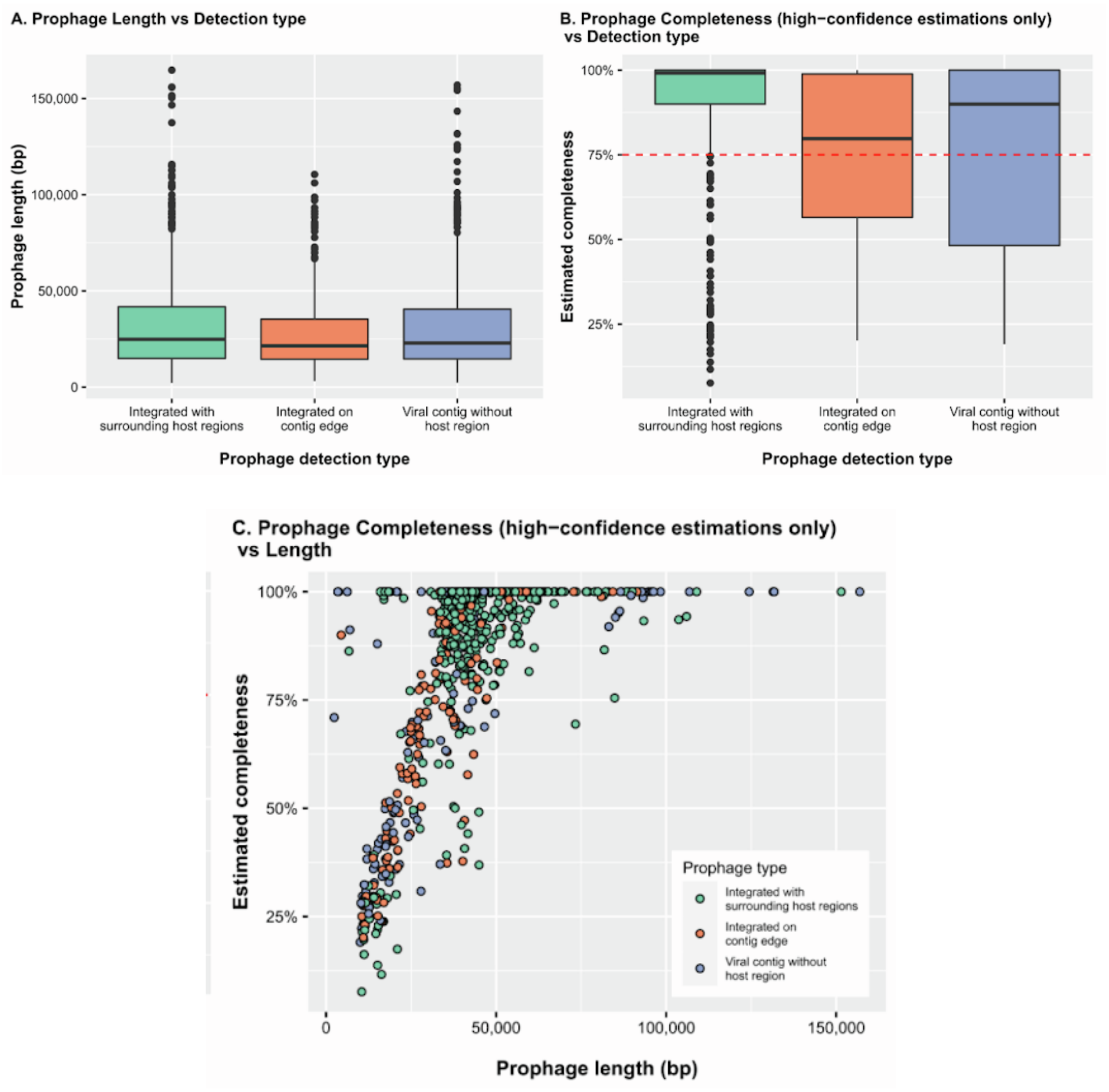

Figure S16. **Host range of individual prophages or VCs across *Actinobacteria* taxa.** For each rank (Genus, Family, Order, Class), prophages (**a**) or vContact2 Viral Clusters (**b**) are classified as infecting a single taxon (blue) or multiple taxa (orange) at this rank. Each time, the proportion of single taxon vs multiple taxa is indicated only for the prophages / VCs that infected multiple taxa at the previous rank. For instance, in panel **a**, the family column shows the distribution of prophages infecting hosts within a single family or hosts across multiple families among the prophages that infected multiple genera. Related to Figure 5.

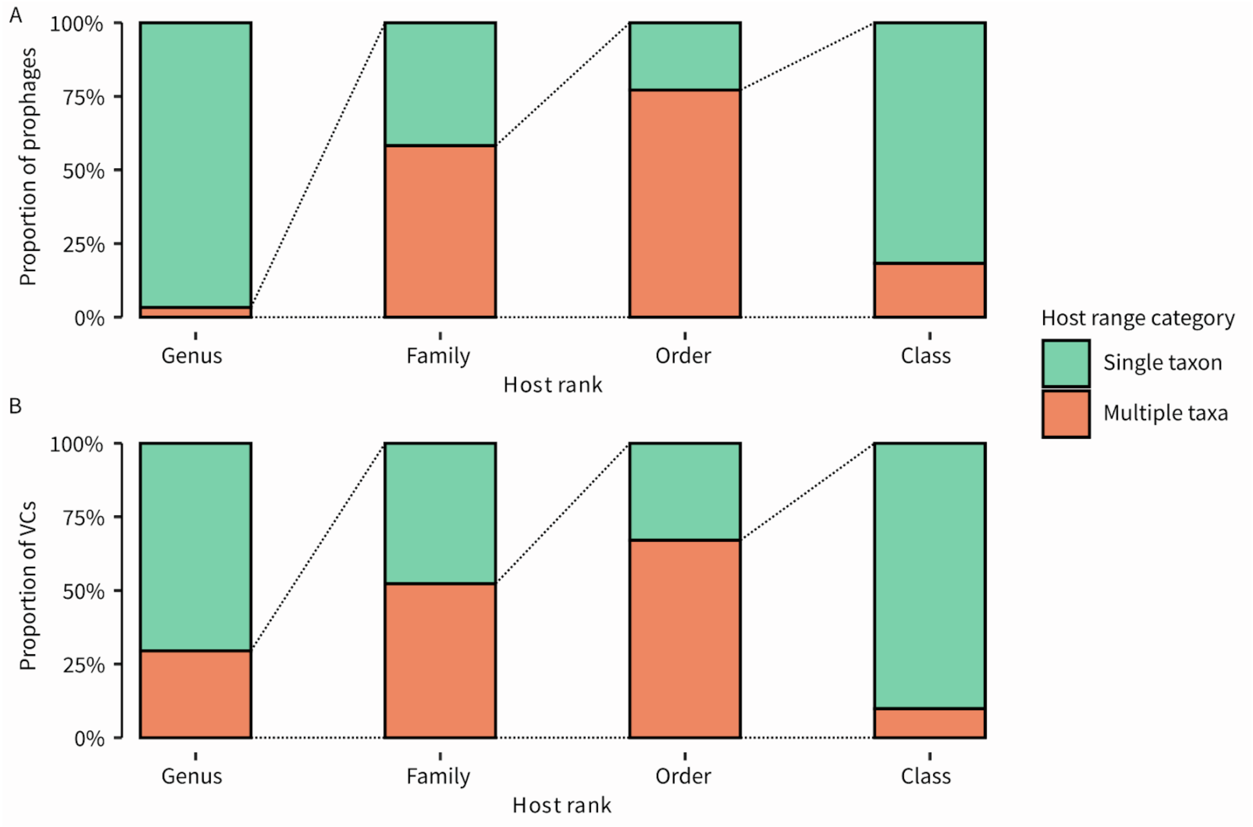

Figure S17. ***Atopobium* prophage encoding Mn/Zn transporters.** For prophages on small (< 80kb) contigs the whole contig is displayed, while only the region surrounding the prophage is displayed otherwise, with coordinates indicated next to the contig ID. Functional annotations were compiled from the IMG and VirSorter2 annotations. Related to Figure 5.

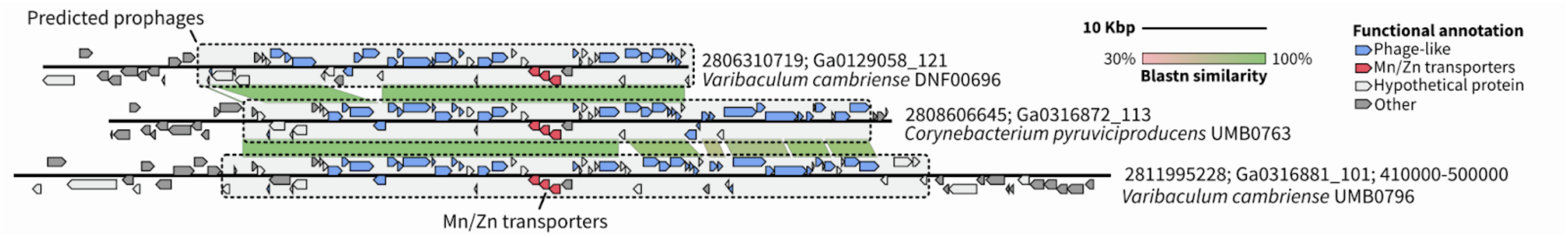
Predicted prophages
2806310719; Ga0129058_121
Varibaculum cambriense DNF00696
2808606645; Ga0316872_113
Corynebacterium pyruviciproducens UMB0763
2811995228; Ga0316881_101; 410000-500000
Varibaculum cambriense UMB0796
Mn/Zn transporters
10 Kbp
30%
100%
Blastn similarity
Functional annotation
Phage-like
Mn/Zn transporters
Hypothetical protein
Other

Supplementary Data

Data S1. Other notable LPD examples of significantly enriched functions summarized in Fig. 2a.

LPD Pfams showing a discordant phylogenetic distribution within a narrow subset of bacterial include domains of unknown function like DUF4300 (PF14133) detected in known pathogenic or host-associated lineages within *Actinobacteria* (e.g., *Corynebacterium* spp., *Trueperella* spp., *Mobiluncus* spp., *Arcanobacterum* spp.) and a few other phyla (e.g., Firmicutes - *Streptococcus* spp., *Clostridioides difficile* str., Bacteroidetes - *Capnocytophaga* spp., *Porphyromonas* spp., Fusobacteria - *Streptobacillus* spp., Spirochaetes - *Treponema denticola* str., etc.) (Figure S4). Although no experimental characterization is reported, the selective taxonomic distribution suggests potential gene transfer between bacteria residing within the same niche (e.g., mammalian oral or digestive system). Furthermore, presence of a signal peptide in most assigned genes suggests a putative role in host-interaction through secretion or surface localization. Many other similar LPD functions (e.g., DUF2781, DUF17197, DUF3060) appear in the host-enriched set and even in the environmental set and are proposed as interesting targets for experimental investigation.

Data S2. Caveats pertaining to BGC prediction and enumeration displayed on Fig 3a.

Given the draft quality and possibly fragmented nature of some of the isolate genomes analyzed here, there is a chance of some inflation of BGC counts – particularly due to fragmentation in regions encoding NRPS and PKS that can span100s of kilobases within a genome, and contain many repetitive functional domains[1]. About 23,453 (28.9%) of total predicted BGCs were found at the contig's end including 1455 (11.9%) of total BGCs from GEBA-Actino genomes generated for this study. Potential mis-assembly or fragmentation (particularly in the case of short read Illumina-sequenced genomes) of large PKS and NRPS regions can impact GCF calculations as well.

REFERENCES

1. Baltz, R.H. (2019). Natural product drug discovery in the genomic era: realities, conjectures, misconceptions, and opportunities. J Ind Microbiol Biotechnol *46*, 281-299. 10.1007/s10295-018-2115-4.
